# Distinct immunomodulation elicited by young versus aged extracellular vesicles in bone marrow-derived macrophages

**DOI:** 10.1186/s12979-024-00472-x

**Published:** 2024-10-21

**Authors:** Dora Livkisa, Tsung-Lin Lee, Wei-Ting Yeh, Manuel S.V. Jaimes, Barbara Szomolay, Chia-Te Liao, David J. Lundy

**Affiliations:** 1 International PhD Program in Biomedical Engineering, College of Biomedical Engineering, Taipei Medical University, 301 Yuantong Road, New Taipei City, 235603 Taiwan; 2 Division of Nephrology, Department of Internal Medicine, Shuang Ho Hospital, Taipei Medical University, New Taipei City, 235603 Taiwan; 3 School of Biomedical Engineering, Taipei Medical University, 301 Yuantong Road, New Taipei City, 235603 Taiwan; 4 Graduate Institute of Biomedical Materials & Tissue Engineering, College of Biomedical Engineering, Taipei Medical University, 301 Yuantong Road, New Taipei City, 235603 Taiwan; 5 Systems Immunity Research Institute, Cardiff University School of Medicine, Cardiff, UK; 6 Division of Infection and Immunity, Cardiff University School of Medicine, Cardiff, UK; 7 Division of Nephrology, Department of Internal Medicine, School of Medicine, College of Medicine, Taipei Medical University, 250 Wuxing Street, Taipei, 110 Taiwan; 8 Taipei Medical University-Research Center of Urology and Kidney, Taipei Medical University, Taipei, 110 Taiwan; 9 Cell Therapy Center, Taipei Medical University Hospital, 250 Wuxing Street, Taipei, 110 Taiwan

**Keywords:** Aging, Ageing, Macrophage, Exosome, Blood plasma, miRNA, Cytokine, Inflammation

## Abstract

**Background:**

Previous research has indicated that extracellular vesicles (EVs) potentially play significant roles in multiple ageing phenotypes. This study uses a factorial experimental design to explore the interactions between circulating EVs and bone marrow-derived macrophages (BMDMs) isolated from young (7–12 weeks) and aged (70–90 weeks) mice.

**Results:**

In this study, plasma EVs from young (Y_EV) and aged (O_EV) mice were isolated and compared based on abundance, size, and miRNA cargo. Compared to some previous studies, we found relatively few differences in EV miRNA cargo between Y_EVs and O_EVs. Young and old EVs were then used to stimulate naïve BMDMs isolated from young (Y_BMDM) and aged (O_BMDM) mice. A panel of five “M1” and six “M2” macrophage markers were used to assess the degree of polarisation. Our results revealed differences in the immunomodulatory effects of Y_EVs and O_EVs in Y_BMDMs and O_BMDMs. Y_EVs induced less pro-inflammatory gene expression, while O_EVs exhibited a more varied impact, promoting both pro- and anti-inflammatory markers. However, neither EV population induced a clearly defined ‘M1’ or ‘M2’ macrophage phenotype. We also report that EVs elicited responses that differed markedly from those induced by whole plasma. Plasma from old mice had strong pro-inflammatory effects on Y_BMDMs, increasing *Il1b*, *Nlrp3* and *Tnfa*. However, O_EVs did not have these effects, supporting current evidence that EVs are a separate component of circulating factors during ageing. More research is needed to elucidate specific factors involved in inflammageing processes.

**Conclusions:**

Our findings reveal age-related differences in EV cargo and function, with young EVs tending to suppress inflammatory markers more effectively than aged EVs. However, this is not straightforward, and EVs often promoted both M1 and M2 markers. These results suggest that EVs are a distinct component of circulating factors and hold potential for therapeutic strategies aimed at mitigating age-related inflammation and immune dysregulation.

**Supplementary Information:**

The online version contains supplementary material available at 10.1186/s12979-024-00472-x.

## Introduction

Extracellular vesicles (EVs) are lipid bilayer-bound vesicles secreted by cells for intercellular communication [1]. EVs carry complex, diverse cargo of proteins, nucleic acids (mRNA, miRNA etc.), lipids and metabolites; each of which can have multiple effects upon target/recipient cells [2, 3]. As such, EVs secreted from organ parenchymal cells, stromal cells, immune cells and circulatory cells form complex communication networks. EV cargo (thus function) is dynamic and components are selectively enriched and secreted based on biological variables [4]. Importantly, EV cargo reflects the physiological condition of the originating tissue, such as age, injury or repair [5, 6]. As such, the contents of circulating EVs can be used as biomarkers [7]. In this study, we utilise plasma EVs from young and aged mice. Use of plasma avoids blood coagulation and release of platelet EVs, which contain their own distinct cargo [8, 9]. Thus, plasma best represents the natural physiological state of the host [10]. Plasma contains circulating EVs released from multiple sites, including liver, lung, adipose tissue and kidney, and these have been shown to change with age; less EVs originate from the brain, while more originate from liver of older mice [7, 11, 12]. Studies have shown that effects of EVs are often attributed to their miRNA cargo, and can be direct (i.e. protection of parenchymal cells or stimulation of endothelial cell angiogenesis) or indirect (i.e. modulation of immune cells, fibroblasts and the inflammatory microenvironment) [13, 14]. 

Immune function and macrophage activity change with increased age [15]. Ageing also affects circulating factors which in turn affect the function of various organs and systems over the lifespan [16]. Several high-profile publications have demonstrated transferrable therapeutic benefits of transfusing young blood into older animals [17–19]. For example, Horowitz and colleagues found that some cognitive benefits of exercise could be recapitulated by administration of plasma from exercised mice to sedentary aged animals, which they attributed to circulating Gpld1 [20]. A recent study showed that young plasma reduced the severity of kidney damage following ischemia/reperfusion (I/R) injury in old mice, which was attributed to anti-inflammatory actions [21]. Similarly, parabiosis with young mice reduces inflammation and the extent of I/R injury in old mice [22]. Circulating EVs from young and old animals have also been explored as biomarkers and in different disease models, and have been found to reflect higher degrees of senescence with ageing [23, 24]. EVs in ageing appear strongly linked to inflammation. For example, EVs from old mice adversely affect outcomes of stroke when administered to younger mice, which was attributed to pro/anti-inflammatory functions [25]. Conversely, a recent study showed that administering EVs from young mice reversed multiple ageing-related pathologies in older mice. This was attributed to differences in EV miRNA cargo [26]. A study of circulating EV miRNAs in aged rats found that age-related changes could be modulated towards a younger phenotype by calorie restriction, again linked to inflammatory signalling and EV miRNA cargo [27]. Calorie restriction itself has been shown to reduce organ fibrosis via modulating miR-21 content of EVs [28]. Lastly, a recent study by our own research group found modulation of circulating EV miRNAs in healthy human volunteers after 72 h of water-only fasting, again linked to anti-inflammatory effects [29]. Taken together, this shows that circulating factors, EVs and their miRNA cargo are modulated by age and appear strongly related to inflammation. Macrophages are early responders after tissue injury and play key roles in subsequent acute and chronic immune responses [30]. Following injury, macrophages secrete growth factors and cytokines which steer vascular permeability, clearance of damaged cells, angiogenesis and fibrosis. Following injury, macrophages play various immunomodulatory roles depending on their phenotype, secreting growth factors and cytokines which alter vascular permeability and influence damaged cell clearance, angiogenesis and fibrosis. Macrophages have been broadly categorised into M1 and M2 types, where M1 are classically-activated, produce reactive oxygen species, and secrete pro-inflammatory cytokines, whereas M2 macrophages participate in tissue remodelling and wound healing and express arginase-1. Furthermore, M2 macrophages can be subdivided into M2a (profibrotic, high TGF-B, CCL-17, low Arg1) M2b (immunomodulatory, high IL-10, IL-6 IL-1b, TNF-a and CD68 and IRF4), and M2c (deactivated, CD206+, IL-10, TGF-B) subtypes [31]. 

Importantly, new evidence indicates that EVs administered systemically, or directly to injured tissues, are taken up by macrophages to a greater extent than by parenchymal cells [3]. Previous studies have also shown that EV miRNAs can induce macrophage polarisation towards pro- or anti-inflammatory phenotypes [32–34]. Together, this suggests that reported benefits of EV transfusions may act via immunomodulation of macrophages. It is also known that young and old bone marrow cells have different immunomodulatory functions [35]. Given the volume of evidence implicating miRNAs as the main drivers of EV effects, we sought to profile plasma EV cargo from young and old mice.

## Aims of this study

Taking these factors into account, we aimed to explore differential effects of young and old EVs on macrophage polarisation using highly controlled in vitro experimental systems. First, we sought to compare plasma EV miRNA cargo, and naïve BMDMs, isolated from young and old mice. Second, we aimed to carry out a factorial experiment using both young/old EV donors and young/old BMDMs to compare age-matched, Y-O and O-Y pairings. Given that macrophages play pivotal roles in both initiating and resolving inflammation, understanding how EVs from different age groups influence macrophage function has implications for age-related diseases and immune dysregulation. The ability of young EVs to reduce inflammatory markers supports their potential as candidates for novel therapeutic interventions aimed at mitigating chronic inflammation and promoting tissue repair in elderly patients.

## Materials and methods

### Animal studies

Bone marrow cells (BMCs) and plasma samples were collected from male C57BL/6 mice, purchased from National Laboratory Animal Center (NLAC), Taiwan. Mice were housed at Taipei Medical University Laboratory Animal Center (non-SPF conditions), with a 12/12 light/dark cycle, *ad libitium* access to water and a standard diet. Young mice were 7–12 weeks old and old mice were 70–90 weeks old at the time of plasma or bone marrow collection. Experiments were carried out with approval from TMU institutional animal care and use committee (protocol numbers LAC-2021-0256 and LAC-2020-0529).

### Blood sampling

Blood was obtained from young and old mice by cardiac puncture and collected into EDTA-anticoagulated (BD, 365974) paediatric-sized collection tubes which were filled with 300–400 µl blood per tube. Best-practice EV isolation guidelines were followed including use of a 21G needle and minimal agitation to avoid platelet activation. The tube was then centrifuged at 1,000 g, for 10 min at 4 ˚C. Supernatant was transferred to a new tube leaving a safety layer above the buffy coat [8]. Plasma was then aliquoted into single-use tubes and stored at -80˚C.

**Plasma EV isolation and characterisation**. EVs were isolated by precipitation (System Bio, EXOQ5TM-1) following the manufacturer protocol for plasma preparation and defibrination. Particle count and diameter was determined by nanoparticle tracking analysis (NTA) using a Malvern Nanosight NS-300 device. The solution was diluted to a concentration of ~ 100 particles per frame and each sample was measured three times for 60 s each. Protein concentration was determined by BCA assay (Thermo, 23227) following standard lab protocols. cryoEM preparation (FEI Vitrobot II) and imaging (FEI Tecnai F-20) was performed by technicians at the Academia Sinica Cryo-EM Facility, located in Academia Sinica, Taipei, Taiwan (ASCEM).

**Isolation and differentiation of bone marrow derived macrophages (BMDMs)**. Bone marrow cells (BMCs) were isolated by following a published method paper [36]. In brief, mouse femurs were removed then flushed with DMEM passing through a 100 μm cell strainer to isolate BMCs. The solution was centrifuged (200x g, 5 min, 4 ˚C) and treated with ammonium-chloride-potassium (ACK) lysing buffer to remove erythrocytes. After washing, cells were resuspended and cultured in DMEM with 10% (v/v) FBS, 1% (v/v) penicillin/streptomycin and 5 ng/ml M-CSF1 at 37˚C in 5% CO_2_. At four days post-isolation, an equal volume of culture medium including 10 ng/ml M-CSF1 was added. This is within the range of other studies using 1 to 25ng/ml [36, 37]. BMDMs are typically analysed at D7 post-isolation [36]. Therefore, at six days post-isolation, cells were washed and medium with EV-depleted FBS (Thermo, A2720801) was used. This was done to minimise interference from bovine protein/miRNA on subsequent experiments and was carried out for all BMDM cultures. We have previously shown that this product is aproximately 99.6% depleted in EVs compared to regular FBS [38]. 

### Flow cytometry

BMCs/BMDMs were suspended at 5 × 10^5^ cells in 0.5 ml DPBS (Gibco, 14190144). Cells were then stained with 0.5 µl near-IR fluorescent reactive dye (Invitrogen, L10119) on ice for 30 min, protected from light. After washing with DPBS, the cells were stained with anti-mouse CD11b (BioLegend, 101217), anti-mouse F4/80 (BioLegend, 123128), anti-mouse CD11c (BD, 558079), and anti-mouse CD206 (BioLegend, 141708) at a concentration of 1:100 in staining buffer on ice for 30 min, protected from light. Following washing with staining buffer, the cells underwent flow cytometry analysis to confirm surface antigen expression. Flow cytometry was performed using an Attune NxT Flow Cytometer (Thermo). Isotype controls were used to determine gates and single-stained cells were used to perform compensation.

### BMDM polarisation and EV treatment

M1/M2 polarisation was induced using 100 ng/ml lipopolysaccharide (LPS) (Sigma, L4391) or 20 ng/ml murine recombinant interleukin 4 (IL-4) (Peprotech, 214 − 14) for 24 h. EVs were provided at 1.5 µg/µl based on our previous study [29]. Precipitation reagent supernatant was confirmed not to affect BMDM gene expression. EV internalisation into BMDMs was visualised by labelling EVs with AlexaFluor-680 using a covalent protein labelling kit (Invitrogen, A20172). Free, unbound, dye was then removed using the provided columns. Labelled EVs were incubated with naïve BMDMs in 35 mm glass petri dishes for 30 min at 37˚C, 5% CO_2_. For imaging, cells were washed thoroughly with PBS, counterstained with DAPI, and images were captured using a Zeiss Stellaris 8 confocal microscope at Taipei Medical University core facility, Taipei, Taiwan. BMDMs incubated with unlabelled EVs were used to set thresholds for non-specific fluorescence, which was minimal.

### RNA extraction and RT-qPCR

mRNA was extracted from BMDMs using isolation kits (Qiagen, 74106 or 74004), reverse transcribed to cDNA using Superscript IV (Thermo, 18-091-050) then amplified using a SYBR green-based master mix (Applied Biosystems, 43-687-08) in a StepOnePlus (Thermo Fisher) thermocycler for 40 cycles. Primers used are shown in **Supplemental Table 1**. All primers were confirmed to lack amplification in the absence of cDNA template. CT values of *Hnrnpa1* were subtracted from each gene to calculate ΔCT and the *mRNA*/*Hnrnpa1* ratio was calculated by 0.5 ^ ΔCT. Log2 fold changes were then calculated for the paired comparisons shown in the figures. Exact statistical comparisons are given in the figure legends.

### miRNA extraction and profiling

miRNA was extracted from plasma EV samples from three young mice and three old mice using qEV RNA extraction kit (IZON, RXT01) which we have previously validated for miRNA isolation and quantification [39]. The lysis buffer was spiked with synthetic miRNAs (Qiagen, 339390) to validate downstream extraction steps. miRNA polyadenylation and reverse transcription was carried out in a StepOnePlus using a miRCURY LNA RT kit (Qiagen, 339340) which also adds spike-in UniSP6 to validate successful cDNA generation. Samples were then validated using a specific quality control kit (Qiagen, 339391) to confirm proper amplification of spike-in miRNAs and housekeeping miRNAs. Following this, the full Rat & Mouse Panel I + II V5 array (Qiagen, 339322, YAMR-312YG-8) was run on a Roche LightCycler 480 in 384-well format. Plate calibration and sample normalisation (Normfinder) were performed using the GeneGlobe platform (Qiagen). All miRNAs were plotted in scatter diagrams. For volcano plot generation, the miRNA expression values were filtered based on Shapiro-Wilk’s test for normality with *p* > 0.05, an assumption for Welch’s t-test, leaving 290 miRNAs. The fold regulation of the 290 miRNAs (as defined in miRCURY) expression values for O_EV vs. Y_EV and the corresponding p-values are shown in the figure. The shapiro.test and t.test R functions were used in R-4.3.3. Samples with mean CT values of < 35, fold changes ≥ 2.0 in old vs. young EV miRNAs, and P values of < 0.05 were considered as significant changes. Data were then exported to Graphpad Prism 10.1 for visualisation. miRNA targets were predicted using microRNA Target Filter in ingenuity pathway analysis (IPA). Results were filtered based on high confidence and involvement in macrophage-specific pathways. For pathway prediction, miRPathDB V2.0 was used.

**Bioinformatics analyses**. Hierarchical clustering was performed for gene expression levels (mRNA/*Hnrnpa1*) for all experimental conditions except for LPS/IL-4 positive controls. Following data standardisation with the scale_rows R function, the clustergram was generated using the Heatmap R function with complete linkage. For multiple pairwise comparisons of selected groups, Kruskal-Wallis test was used, followed by Dunn’s test with Bonferroni correction to identify which pairs of conditions are different (kruskal_test and dunn_test R functions, R version 4.3.2).

### Other software and statistical analyses

Raw data were collected in Microsoft Excel and graphs were made using Graphpad Prism 10.1 (Mac). Flow cytometry data were analysed using FlowJo 10.1. Comparisons of specific groups and the exact statistical tests used are indicated in figure legends. Each data point on graphs refers to a separate sample and all error bars show the standard error of the mean unless stated otherwise.

## Results

### Characterisation of EVs from young and old mice

**Fig. 1:**
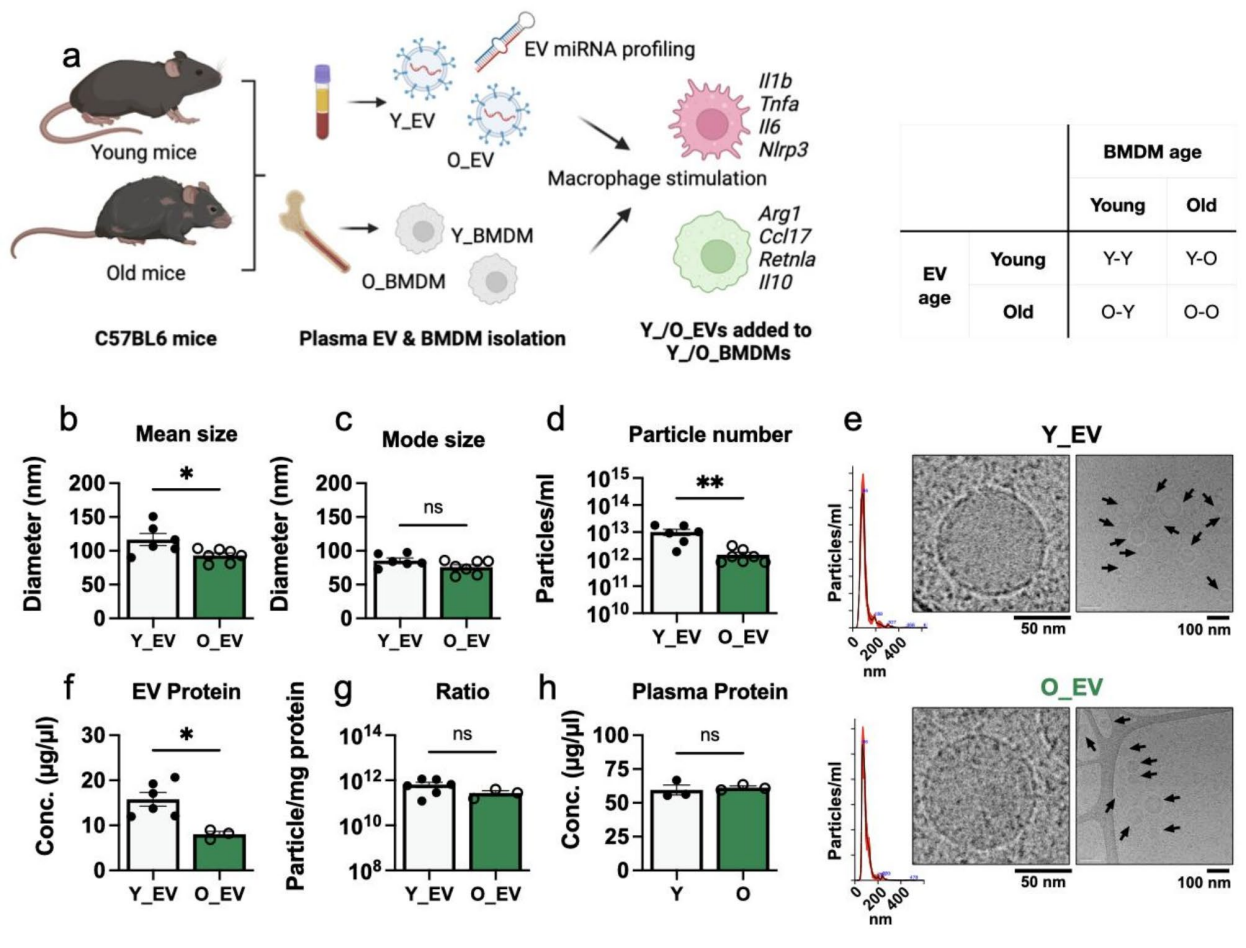
Comparison of extracellular vesicles isolated from young and old mouse plasma (**a**) Schematic diagram of project experimental design. Y_EV = young mouse plasma extracellular vesicle, O_EV = old mouse plasma extracellular vesicle, Y_BMDM = young mouse bone marrow-derived macrophage, O_BMDM = old mouse bone marrow derived-macrophage. The table shows the factorial experimental design. (**b**) Mean diameter of Y_EV and O_EVs, determined by NTA. (**c**) Mode diameter of Y_EVs and O_EVs, determined by NTA. (**d**) Total particle concentration of Y_EVs and O_EVs, determined by NTA. (**e**) High magnification and low magnification cryoEM images of Y_EV and O_EV isolates, with representative NTA peaks. EVs are indicated by arrows in the low magnification image. (**f**) Total protein concentrations of Y_EV and O_EV isolates. (**g**) Particle: protein ratio for Y_EV and O_EV isolates. (**h**) Total protein concentration of young (Y) and old (O) mice plasma. Each data point represents EVs isolated from a separate animal. Bar heights represent the mean and error bars show standard error of the mean. Y_EVs were compared to O_EVs by unpaired two-way *t*-test. ns = not significant (*P* > 0.05), * = *P* ≤ 0.05, ** = *P* < 0.001

The overall experimental design of this project is shown in Fig. 1a. Mouse plasma EVs from young mice (Y_EVs) and old mice (O_EVs) were isolated and first characterised by nanoparticle tracking analysis (NTA). The mean diameter (Fig. 1b) of Y_EVs (from 6 mice) was 116.6 nm and O_EVs (from 7 mice) were 93.0 nm, which was statistically significant (*P* = 0.026, unpaired t-test). The mode diameter (Fig. 1c) of O_EVs was also smaller on average (75.8 vs. 85.5 nm) but this was not statistically significant (*P* = 0.099). We also found that the total particle concentration (Fig. 1d) was lower for O_EVs. These findings agree with previously published data of circulating EVs from old and young mice [23, 25, 40]. NTA showed single peaks at 80–120 nm for both Y_EV and O_EV isolates, confirming a typical EV-sized population. To confirm that the measured particles were indeed EVs, we used cryoEM (Fig. 1e) to inspect the morphology. CryoEM showed multiple spherical, ~ 100 nm vesicles with bi-layered membranes, typical of EVs. Low magnification and high magnification images are shown. Though EV proteins were not measured in this study, this EV isolation method is well-validated by our group and others, and EVs isolated by this method contain CD81, CD9, CD63, HSP70, TSG-101 and other EV markers [29, 41, 42]. The total protein concentration of O_EV isolates was significantly (*P* = 0.038) lower than Y_EVs (Fig. 1f), corresponding to the lower number of particles (Fig. 1d). The particle to protein ratio (Fig. 1g) was no different between Y_EV and O_EV samples, indicating that the isolations were of similar purity [43]. To confirm this, we measured the protein concentration of the original young and old mouse plasma, as shown in Fig. 1h. The results shows that young and old mice all had plasma protein concentrations within a normal range of 59 to 63 µg/µl, and were not significantly different (*P* = 0.67, unpaired t-test). Therefore, taken together, these results indicate that the reduction in particle count likely reflects a true reduction in circulating EV concentration in older mice and there was no different in the purity of EVs used in subsequent experiments.

## Isolation and differentiation of young and old bone marrow-derived macrophages

**Fig. 2:**
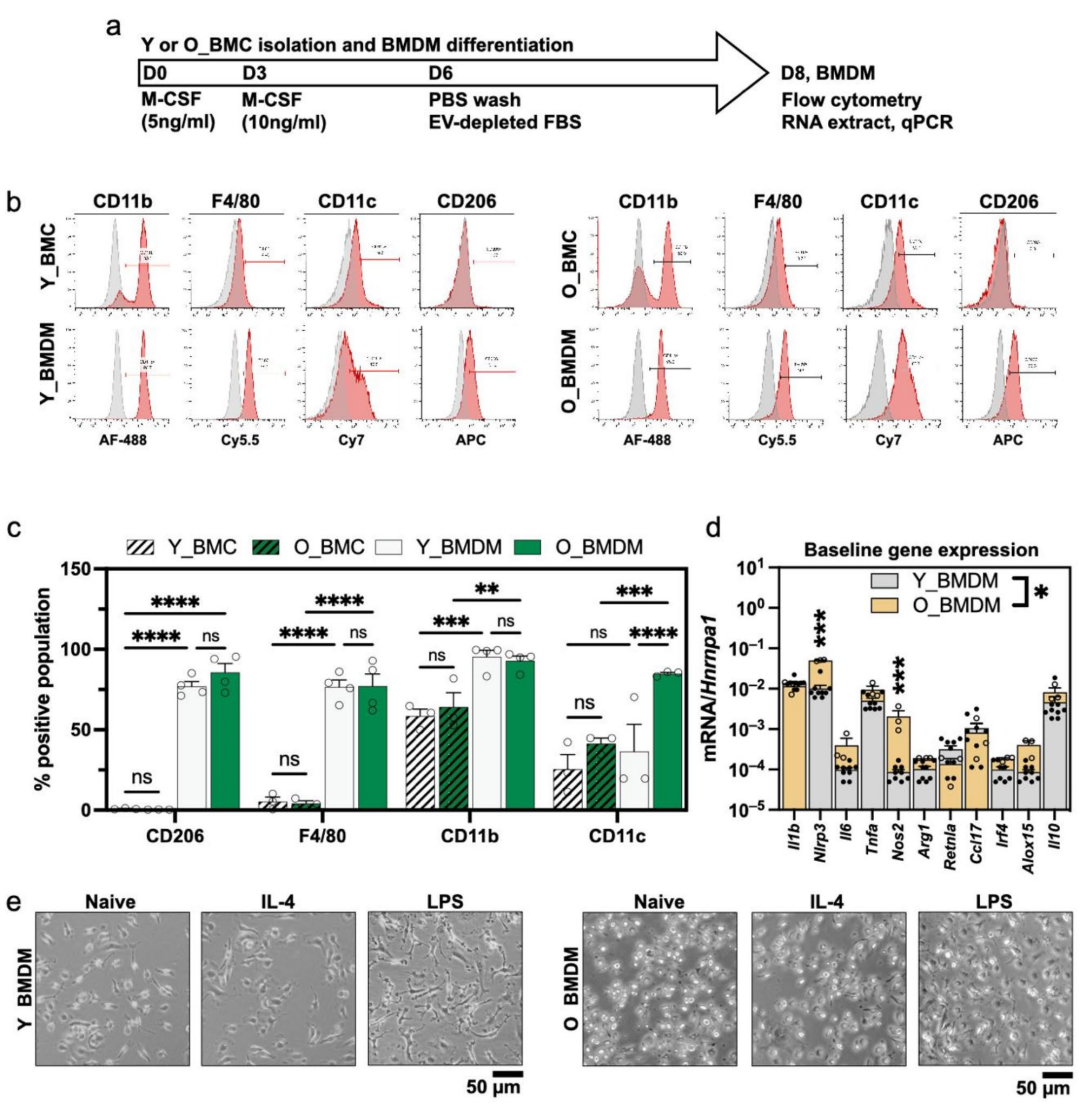
Comparison of bone marrow derived macrophages (BMDMs) isolated from young and old mice (**a**) Experimental timeline. Bone marrow-derived cells (BMCs) were isolated, differentiated with M-CSF, washed and switched to EV-depleted FBS medium at D6, and analysed at D8. (**b**) Representative flow cytometry histograms of young (Y_) and old (O_) mouse BMCs at D1 and BMDMs at D8. Positive % for F4/80 (Y axis) and CD11b (X axis) is shown. *N* ≥ 3 independent batches were analysed. The grey histogram shows isotype control samples. (**c**) Flow cytometry showing positive population (X axis) for CD206, F4/*), CD11b and CD11c. *N* = 3 independent batches were analysed. Samples were compared by two-way ANOVA. (**d**) Quantification of macrophage markers by RT-qPCR in naïve Y_ (*n* = 9) and O_ BMDMs (*n* = 3) at D8. Expression was normalised to *Hnrnpa1* and Y_BMDMs and O_BMDMs were compared by one-way ANOVA, with Tukey’s post-test, indicated by *. (**e**) Example images of Y_/O_BMDMs in naïve, IL-4 or LPS-polarised conditions. Data points show samples from separate mice. Bar heights show the mean, error bars show the standard error of the mean. * = *P* ≤ 0.05, ** = *P* < 0.01, *** = *P* ≤ 0.001, **** = *P* ≤ 0.0001

Next, we isolated bone marrow cells (BMCs) from young and old mice and differentiated them into bone marrow-derived macrophages (BMDMs); termed Y_BMDM and O_BMDM respectively. A schematic timeline is shown in Fig. 2a. Cells were washed and switched into medium containing EV-depleted FBS at D6 to mimic the protocol of upcoming experiments. First, we validated our BMC isolation and BMDM differentiation protocols using flow cytometry (*n* = 3–4 mice per group) to examine surface markers of Y_BMCs or O_BMCs (at D1) and Y_BMDMs or O_BMDMs (at D8). Example histograms for CD11b, F4/80, CD11c and CD206 are shown in Fig. 2b and quantified results are shown in Fig. 2c. The results showed that freshly isolated Y_BMCs and O_BMCs had low F4/80^+^ (< 10%) and CD206^+^ (< 1%) populations, 65–70% positivity for pan-myeloid marker CD11b and 25–50% positivity for CD11c. None of the markers were statistically significantly different between Y_BMCs and O_BMCs. After differentiation to BMDMs, F4/80 increased to > 75%, CD11b increased to > 80% and CD206 (mature macrophage and also M2 marker) increased to > 80% after differentiation. Together, these data indicate successful differentiation of BMCs to BMDMs, and there were no differences in F4/80, CD206 or CD11b between Y_/O_BMCs or Y_/O_BMDMs. However, CD11c, a marker of M1-type macrophages, was higher in freshly-differentiated naïve O_BMDMs (mean 85.5%) than Y_BMDMs (mean 39.7%, *P* = 0.0017) [44, 45]. 

Lastly, we compared basal gene expression levels of genes in naïve Y_BMDMs and O_BMDMs using RT-qPCR to detect changes associated with the M1 or M2 phenotype (Fig. 2d). *Hnrnpa1* has been previously reported as suitable for normalisation of BMDM gene expression [46]. Our preliminary testing also confirmed that *Hnrnpa1* showed stable expression with an average cycle threshold (CT) of 24.1 and coefficient of variation (CV%) of 3.6%, whereas *Gapdh* showed more variability (mean CT 26.1, CV% 8.8%) and another candidate *Stx5a* had much lower expression (mean CT 28.5, CV% 4.0%) (**Supplemental Fig. 1a**). *Hnrnpa1* CT values did not differ between Y_BMDMs and O_BMDMs, and were unaffected by LPS, IL-4 or EV treatments (**Supplemental Fig. 1b**). Thus, *Hnrnpa1* was used as a baseline for normalisation of all further experiments. In the naïve state, O_BMDMs expressed 5-fold higher *Nlrp3* (*P* ≤ 0.001) and 24-fold higher *Nos2* (*P* ≤ 0.0001) than Y_BMDMs, as shown in Fig. 2d. This suggests that naïve O_BMDMs had a slightly higher pro-inflammatory baseline than Y_BMDMs, in agreement with higher CD11c^+^ population measured by flow cytometry. All naïve BMDMs expressed low amounts of *Il6*, *Tnfa*, *Arg1*, *Irf4*, *Retnla* and *Alox15*, indicating that neither were polarised towards either an M1 or M2-like phenotype. Images of BMDMs before and after polarisation are shown in Fig. 2e. Naïve cells and IL-4-stimulated BMDMs were mostly round with occasional protrusions, whereas LPS-stimulated cells were a highly elongated, spindle shapes with multiple protrusions, typical of M1 macrophages. Flow cytometry for CD206 confirmed successful M2 polarisation for both Y_BMDMs and O_BMDMs (**Supplemental Fig. 1c**).

## Examination of young and old mouse EV miRNA cargo

**Fig. 3:**
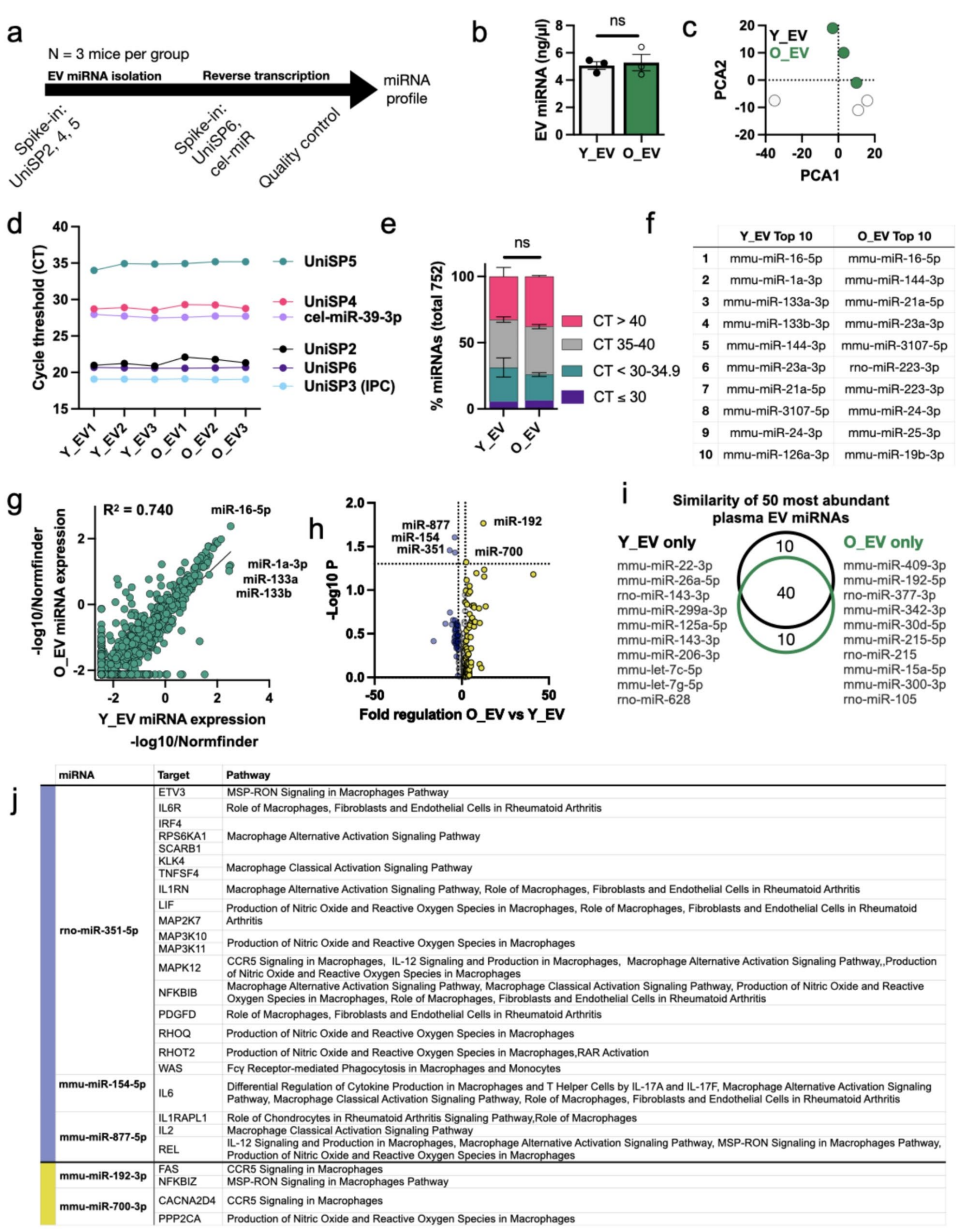
Comparison of miRNA cargo isolated from young and old mouse plasma EVs (**a**) Schematic diagram showing timeline of miRNA isolation, quality control checks and qPCR-based array. Plasma EVs from three mice per group were analysed. (**b**) Total miRNA yield from Y_EVs and O_EVs. Samples were compared by unpaired t-test. (**c**) Principal components analysis (PCA) of Y_EV (silver) and O_EV (green) miRNA cargo. (**d**) Cycle threshold (CT) values for spike-in miRNAs UniSP6 (pre-reverse transcription), UniSP2, 4 and 5 representing high, medium and low expressed miRNAs, and UniSP3 inter-plate calibration (IPC). cel-miR-39-3p is also included. (**e**) Percentage of miRNAs with high (CT ≤ 30), medium (30-34.9), low (35–40) and absent expression out of 752 assayed miRNAs. No significant differences in populations were detected. (**f**) The most abundant miRNAs in Y_EV and O_EV samples, listed from highest to lowest. (**g**) Scatter diagram of Y_EV (x axis) vs. O_EV (Y axis) miRNA expression levels normalised using NormFinder. A correlation value is shown, and some highly abundant miRNAs are annotated. (**h**) Volcano plot showing statistical significance (Y axis) against fold-change (X axis). The Y axis line represents a P value of 0.05 and X axis lines show fold changes of + 2.0 or -2.0. Significantly different miRNAs are annotated. (**j**) Venn diagram showing overlap of the 50 most abundant miRNAs in Y_EV and O_EV samples. The miRNAs found in only one population are annotated. Target prediction of differentially-regulated miRNAs relevant to macrophages

Next, we sought to compare EV miRNA cargo. Previous studies, using alternative EV isolation and miRNA comparison methods, have shown changes in circulating miRNAs and plasma EV miRNAs in young and old mice and rats [23, 26, 27]. In our study, EV miRNA was extracted from three mice per group, subjected to quality control analyses and then analysed using a qPCR-based rodent-specific panel to specifically detected 752 known miRNAs, as shown in Fig. 3a. There was no difference in total miRNA yield between Y_EVs and O_EVs (Fig. 3b). Principal component analysis of the entire profile (Fig. 3c) showed moderate clustering of Y_EV and O_EV samples. All samples showed stable expression of spike-in cel-miR-39-3p, and expected high/medium/low expression of UniSP2/4/5, as shown in Fig. 3d. UniSP3, used to calibrate across different miRNA qPCR plates, was extremely consistent. This indicates successful and consistent miRNA isolation, reverse transcription and linear amplification of all samples. The results of the panel (Fig. 3e) found that 25.7% (193 miRNs) and 19.7% (148 miRNAs) of Y_EV and O_EV miRNAs had cycle threshold (CT) values below 35. These proportions were not significantly different between Y_EVs and O_EVs. Looking at the most abundant miRNAs (CT ≤ 30.0) 40 were found in Y_EVs and 47 were found in O_EVs. The most abundant 10 miRNAs in Y_EVs and O_EVs are shown in Fig. 3f. Abundant miRNAs in both samples were mmu-miR-16-5p, mmu-miR-144-3p, mmu-miR-21a-5p, mmu-miR-23a-3p, miR-1a-3p and mmu-miR-3107-5p (also termed miR-486b-5p). These miRNAs are commonly detected in plasma EV samples [47–49]. Many have also been previously linked to macrophage polarisation. miR-16-5p, the most abundant miRNA in O_EVs, has been previously shown to decrease M1 marker gene expression in RAW264.7 cells [50]. Similarly, miR-144, the second most abundant O_EV miRNA, has been shown to negatively regulate inflammatory responses *via* suppressing TRAF6 which is known to promote the M2 macrophage phenotype [51]. A scatter plot of normalised miRNA expression levels (Fig. 3g) showed that overall miRNA expression levels were very similar between Y_EV and O_EV miRNAs, with a high degree of correlation (R^2^ = 0.74). Five miRNAs met our criteria for differential expression in O_EVs compared to Y_EVs, as shown in the volcano plot (Fig. 3h). The overlap between the top 50 most abundant miRNAs in Y_EVs and O_EVs are illustrated by Venn diagram (Fig. 3i), again showing a high degree of overlap (40/50 miRNAs present in both populations). Statistically comparing miRNA expression in O_EVs versus Y_EVs we found significant decreases in mmu-miR-877-5p, miR-154-5p and rno-miR-351-5p, and significant increases in mmu-miR-192-3p and mmu-miR-700-3p. These differentially expressed miRNAs are shown in Table 1.

**Table 1:** Differentially abundant miRNAs between O_EVs and Y_EVs

miRNA ID	*p*-value	Fold regulation
mmu-miR-192-3p	0.0171	12.3234418
mmu-miR-877-5p	0.0249	-4.0746232
mmu-miR-154-5p	0.0350	-6.9162979
rno-miR-351-5p	0.0371	-3.8548285
mmu-miR-700-3p	0.0479	2.5256709

These five miRNAs were used for microRNA Target Filter in IPA to identify potential target genes. Results were filtered based on murine-specific high confidence or experimental confirmation and involvement in macrophage-specific pathways (Fig. 3j). In particular, rno-miR-351-5p, reduced in O_EVs, has multiple targets related to macrophage alternative and classical activation, nitric oxide and reactive oxygen species production. miRNAs increased with age, miR-1923p and miR-700-3p target genes involved in CCR5 signalling and nitric oxide/reactive oxygen species. Out of the top 10 miRNAs, 8 miRNAs had targeting information available for old mice and for young mice. The microRNA Target Filter results in IPA were filtered based on murine-specific high confidence or experimental observation, targeting 749 mRNAs for old and 718 mRNAs for young, which were used for PANTHER overrepresentation test. Significant pathways (FDR < 0.05) are shown in **Supplementary Fig. 3**. Shared pathways included those related to responses to oxidative stress, TGF-beta signaling, FGF signaling, inflammation, cytokines and chemokines. O_EV miRNA specific pathways included those related to Ras, p38, interferon-gamma and EGF receptor signaling, demonstrating that the Y_EV and O_EV miRNA cargos are distinct and have different activities.

Interestingly, our EV miRNA profiling results were quite different to a previous analysis of old and young mouse plasma EVs by Alibhai and colleagues [23]. Their study found significant increases in miR-21, miR-145, miR-146a, miR-223 and let-7a in EVs from aged mouse plasma compared to young mice. In our study, most these miRNAs were also detected in plasma EVs, but they were not significantly different between young and old mice. A summary of miRNAs from other studies compared to our own is shown in **Supplementary Table 2.** The main ageing-related miRNA identified in their study, miR-146a, trended towards higher expression in O_EVs (1.36-fold) but was not significant (*P* = 0.68) in our data. Alibhai and colleagues also found increases in plasma EV miR-22 and reductions in plasma EV miR-455, let-7i, miR-200c-3p and miR-199b in aged mice. These were not statistically significantly different in our experiment.

### Responses of young and old BMDMs to known polarising stimuli

**Fig. 4:**
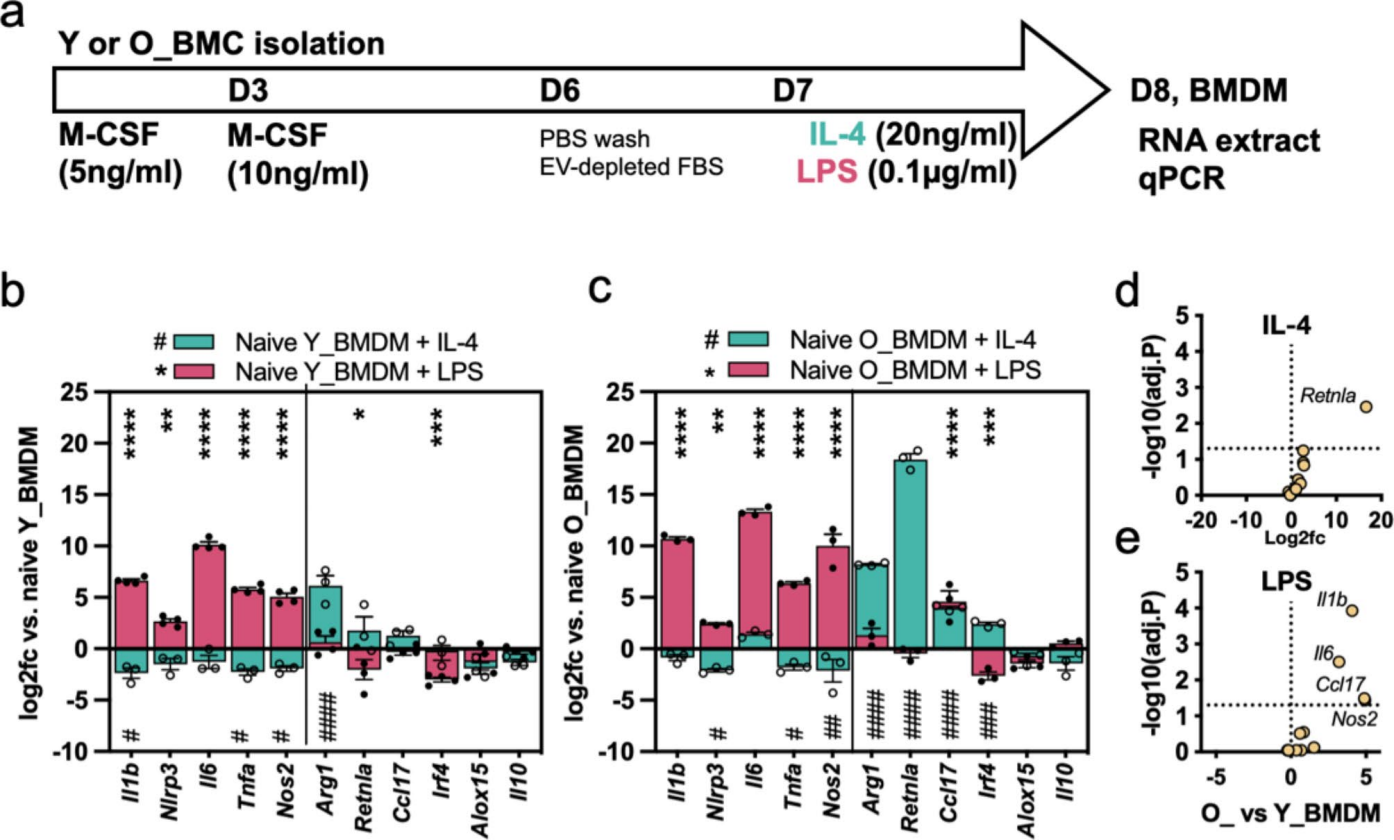
Response of young and old mouse BMDMs to known polarising stimuli (**a**) Experimental timeline. At D7 BMDMs were stimulated with IL-4 or LPS. At D8 the BMDMs were assessed by qPCR. (**b**) Log2fc of gene expression changes in Y_BMDMs following treatment with IL-4 (teal colour bars) or LPS (pink bars), compared to naive (PBS-treated) BMDMs which were assigned a log2fc of zero. Significant changes after LPS are shown by an asterisk (*) and changes with IL-4 are shown by hash (#). Samples were compared to naïve cells by 2-way ANOVA with Dunnett’s multiple comparison correction. (**c**) Log2fc of gene expression changes in O_BMDMs following treatment with IL-4 or LPS. (**d**) Volcano plot showing comparison between O_ and Y_BMDMs treated with IL-4. The X axis shows log2-fold changes and the Y axis shows P values (-log10 transformed to enable fitting to the graph). The dotted line on the Y axis indicates *p* = 0.05 and those above the line are considered significant. O_BMDM and Y_BMDM results were compared by unpaired t-test with Holm-Sidak correction for multiple comparisons. (**e**) Volcano plot showing comparison between O_ and Y_BMDMs treated with LPS. Each data point shows a separate BMDM sample, bar heights show the mean, and error bars show the standard error of the mean. * = *P* ≤ 0.05, ** = *P* ≤ 0.01, *** = *P* ≤ 0.001, **** = *P* ≤ 0.0001

Next, we compared how Y_BMDMs and O_BMDMs responded to known polarising stimuli; using LPS to induce pro-inflammatory M1-type polarisation and IL-4 to induce M2-type polarisation. A schematic illustration of the experimental design is shown in Fig. 4a. In Y_BMDMs, LPS significantly increased *Il1b* (100-fold), *Nlrp3* (6-fold), *Il6* (1,129-fold), *Tnfa* (55-fold) and *Nos2* (33-fold) expression, and significantly decreased *Retnla* (-4-fold) and *Irf4* (-8-fold) compared to naïve cells (Fig. 4b). On the other hand, IL-4 significantly decreased *Il1b (-5-fold)*,* Tnfa* (-4.7-fold) and *Nos2* (-4-fold) and increased M2 marker *Arg1* (71-fold). All of these are expected changes following LPS/IL-4 exposure and demonstrate successful BMDM isolation and polarisation. O_BMDMs responded to LPS similarly as Y_BMDMs, showing large increases of *Il1b*, *Nlrp3*, *Il6*, *Tnfa* and *Nos2*, as well as reduced *Irf4* after LPS stimulation (Fig. 4c). O_BMDM M2 markers responded more strongly to IL-4 showing statistically significant increases in *Arg1* (294-fold), *Retnla* (> 350,000-fold), *Ccl17* (16-fold) and *Irf4* (5-fold) expression. Interestingly, *Ccl17* was also increased by both IL-4 and LPS in O_BMDMs. Directly comparing the response of O_BMDMs vs. Y_BMDMs to IL-4 (Fig. 4d) and LPS (Fig. 4e), visualised as volcano plots, showed that O_BMDMs showed greater increases in expression of pro-inflammatory *Il1b*,* Il6* and *Nos2* following LPS stimulation, and higher *Retnla* after IL-4 stimulation. In summary, Y_BMDMs and O_BMDMs both showed expected changes in gene expression and successful polarisation following LPS or IL-4 stimulation; but O_BMDMs showed greater magnitudes of change, particularly in terms of higher M2 marker expression in response to IL-4. Flow cytometry for CD206 was also used to confirm macrophage M2 polarisation. The CD206^+^ population was downregulated by LPS and upregulated by IL-4 in both Y_BMDMs and O_BMDMs compared to naïve cells, as expected (**Supplemental Fig. 1c**). Again, O_BMDMs showed the highest CD206^+^ population after IL-4 treatment. Together, these data demonstrate that the BMDMs used in these experiments responded normally to standard polarising stimuli.

### Responses of young and old BMDMs to young and old mouse plasma

**Fig. 5:**
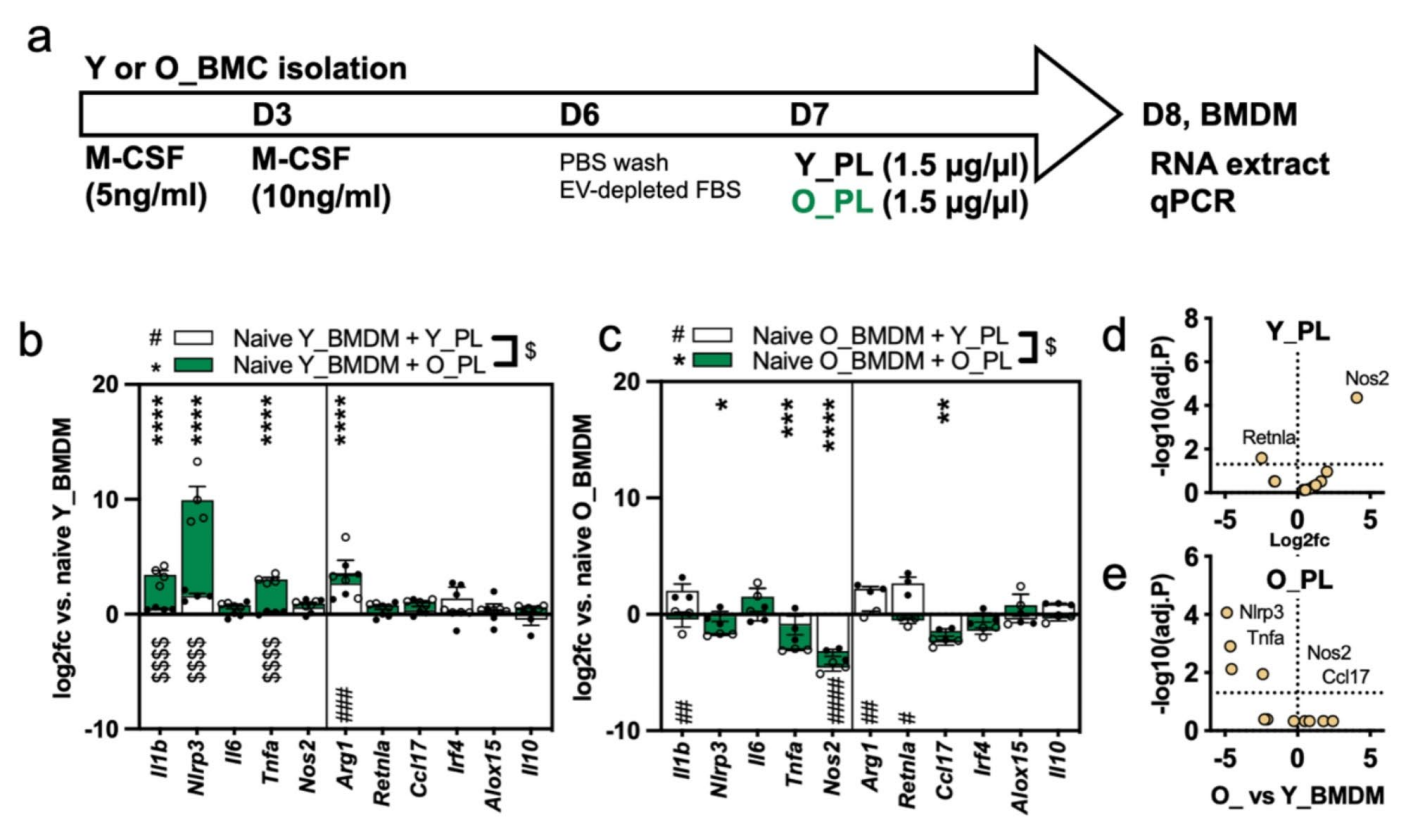
Response of young and old BMDMs to young and old mouse plasma (**a**) Experimental timeline. At D7 BMDMs were stimulated with young mouse plasma (Y_PL) or old mouse plasma (O_PL) for 24 h. (**b**) Log2fc of gene expression changes in Y_BMDMs following treatment with Y_PL (white bars) or O_PL (green bars), compared to naive (PBS-treated) Y_BMDMs. Significant changes after Y_PL are shown by an asterisk (*) and changes with O_PL are shown by hash (#). Comparisons of Y_PL vs. O_PL are shown by $. (**c**) Log2fc of O_BMDMs treated with Y_PL or O_PL. (**d**) Volcano plot showing comparison of O_ and Y_BMDMs treated with Y_PL. (**e**) Volcano plot showing comparison of O_ and Y_BMDMs treated with O_PL. Data points show separate BMDM samples, bar heights show the mean and error bars show the standard error of the mean. For all comparisons * = *P* ≤ 0.05, ** = *P* ≤ 0.01, *** = *P* ≤ 0.001, **** = *P* ≤ 0.0001

Next, we examined the effects of young (Y_PL) and old (O_PL) whole plasma on naïve macrophages, as shown in Fig. 5a. The whole plasma contains carrier proteins, lipoproteins, free cytokines, hormones, immune factors and EVs, although EVs make up only a small percentage of the total plasma protein [8]. The results from Y_BMDMs (Fig. 5b) clearly showed that O_PL induced large increases of M1-associated *Il1b*, *Nlrp3* and *Tnfa*. M2 marker *Arg1* was also increased (12-fold), but no other M2 markers were significantly changed. Y_PL had no significant effects on any M1 markers in Y_BMDMs, but similarly increased *Arg1* (6-fold). Together, these data indicate that O_PL had much stronger pro-inflammatory effects on Y_BMDMs than Y_PL. O_BMDMs (Fig. 5c) responded very differently to plasma than Y_BMDMs. Aside from a small increase in *Il1b* (4-fold), Y_PL caused broadly anti-inflammatory changes; decreasing *Nos2* (-10-fold), and increasing M2 markers *Arg1* (4.6-fold) and *Retnla* (6-fold). O_PL also had opposite effects on O_BMDMs than Y_BMDMs, decreasing pro-inflammatory *Nlrp3* (-3-fold), *Tnfa* (-8-fold) and *Nos2* (-24-fold). The differences between the response of Y_BMDMs and O_BMDMs to Y_PLs and O_PLs are shown in Fig. 5d and e respectively. Together, these data show that O_PL is pro-inflammatory to Y_BMDMs, but was mostly anti-inflammatory to O_BMDMs. On the other hand, Y_PL was mostly neutral to Y_BMDMs and O_BMDMs. Additionally, immunosuppressive and stimulative markers *Tgfb1* and *Pdl1* were examined in O_BMDMs treated with IL-4 or O_PL, as shown in **Supplemental Fig. 4**. Both markers were expressed, and O_PL induced a slight (0.8-fold) reduction in *Pdl1* expression.

### Responses of young and old BMDMs to young and old mouse plasma EVs

**Fig. 6:**
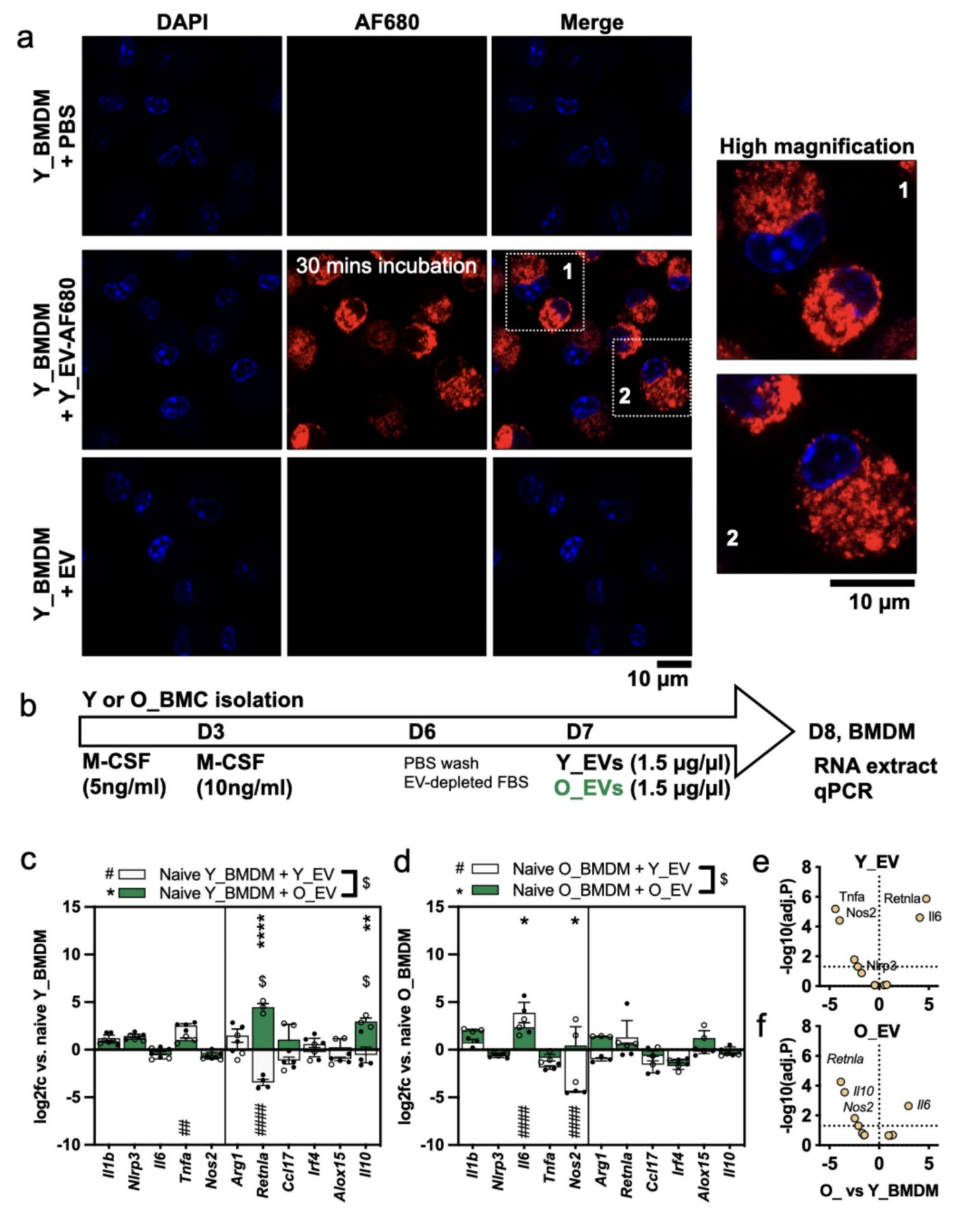
Response of young and old BMDMs to young and old mouse plasma EVs (**a**) Representative confocal microscope images of EV internalisation by BMDMs. The example shows Y_BMDMs after 30 min incubation with AF680-labelled Y_EVs. The right column shows the two annotated inset areas at higher magnification. 10 μm scale bars are shown. (**b**) Experimental timeline of EV-induced BMDM polarisation experiment. (**c**) Log2fc of gene expression changes in Y_BMDMs following treatment with Y_EVs (white bars) or O_EVs (green bars), compared to naive (PBS-treated) BMDMs. Significant changes after Y_EVs are shown by a hash (#) and changes with O_EVs are shown by an asterisk (*). Comparisons of Y_EVs vs. O_EVs are shown by $. (**d**) Log2fc of gene expression changes in O_BMDMs following treatment with Y_EVs or O_EVs. (**e**) Volcano plot showing comparison between O_ and Y_BMDMs treated with Y_EVs. The Y axis shows -log10-transformed P values (to enable plotting) and the X axis shows log2 fold change. The dotted line on the Y axis indicates *p* = 0.05 and those above the line are considered significant. (**f**) Volcano plot showing comparison between O_ and Y_BMDMs treated with O_EVs. Bar heights show the mean and error bars show the standard error of the mean. Data points show separate BMDMs samples. For all comparisons * = *P* ≤ 0.05, ** = *P* ≤ 0.01, *** = *P* ≤ 0.001, **** = *P* ≤ 0.0001

Lastly, we examined the effects of Y_EVs and O_EVs added to naïve Y_BMDMs and O_BMDMs at D7. EVs were given at a dose which represents 2.5% of the plasma protein concentration. First, we confirmed EV internalisation using Y_EVs and naïve Y_BMDMs as an example. The results (Fig. 6a) clearly showed internalisation of AlexFluor-680 labelled EVs into the macrophage cytoplasm after 30 min incubation, followed by thorough washing to remove EVs which were not internalised. This is expected, since BMDMs are known to very rapidly take up EVs by membrane fusion, endocytosis and phagocytosis. For measuring polarisation, BMDMs were treated with EVs for 24 h, as shown in Fig. 6b. In Y_BMDMs (Fig. 6c), a few significant changes in gene expression were noted after the addition of EVs. Y_EVs increased *Tnfa* (5.8-fold) and decreased *Retnla* (11-fold) expression, indicating mild pro-inflammatory stimulation, whereas O_EVs increased *Il10* (8-fold) and *Retnla* (22-fold) indicating anti-inflammatory stimulation. Both Y_EVs and O_EVs induced small average increases in *Il1b* and *Nlrp3*, but these were not statistically significant (*P* = 0.23 and 0.12 respectively). In O_BMDMs (Fig. 6d**)**, both Y_EVs and O_EVs significantly raised *Il6* (14-fold and 5-fold respectively) but Y_EVs significantly reduced *Nos2* (-21-fold). Neither Y_EVs nor O_EVs produced any changes in M2 markers in O_BMDMs. Statistically comparing the effects of Y_EVs against effects of O_EVs (indicated by $ in the figure), *Il10* and *Retnla* were significantly different in Y_BMDMs and no differences were observed in O_BMDMs. Differences between responses of Y_BMDMs and O_BMDMs are presented in Fig. 6e and f. Together, these results indicate that both Y_EVs and O_EVs could modulate BMDM gene expression, but neither EV type induced a distinct M1 or M2-like signature. O_EVs tended towards inducing more inflammatory markers in naïve Y_BMDMs, but also increased *Il10*. This could potentially represent an M2b phenotype which expresses inflammatory markers alongside *Il10*.

Notably, the results using isolated EVs were very different to those obtained using whole plasma (Fig. 5). In particular, O_EVs did not have the same pro-inflammatory effects as their originating O_PL. This highlights how the circulating EV component possesses its own activities and shows that EVs may be advantageous for inducing anti-inflammatory outcomes. Future experiments could evaluate EV-depleted plasma fractions using size-exclusion chromatography.

### Effect of EV pre-treatment on LPS-induced polarisation

We considered that in the in vivo environment, macrophages would be in the presence of circulating factors **prior** to any pro-inflammatory stimulus, which may dictate their response. Therefore, we pre-treated Y_BMDMs with Y_EVs or O_EVs for 24 h before adding a low concentration of LPS to induce pro-inflammatory stimulus, as shown in **Supplemental Fig. 2a**. The results (**Supplemental Fig. 2b**) showed that LPS induced typical M1-type polarisation, regardless of the EV pre-treatment, and there were no significant differences between effects of Y_EVs or O_EVs. However, directly comparing EV pre-treated Y_BMDMs against non-EV pre-treated Y_BMDMs in the presence of LPS (**Supplemental Fig. 2c**) showed that both EV groups increased *Il10* and *Retnla* and decreased *Il6.* Interestingly, there was no significant difference between pre-treatment with Y_EVs or O_EVs. Though neither EV population could prevent LPS-induced M1 polarisation, both reduced the magnitude of changes compared to non-pre-treated LPS-stimulated BMDMs. This suggests that plasma EVs have broadly anti-inflammatory functions, regardless of donor age.

### Summary of BMDM responses to extracellular vesicles

**Fig. 7:**
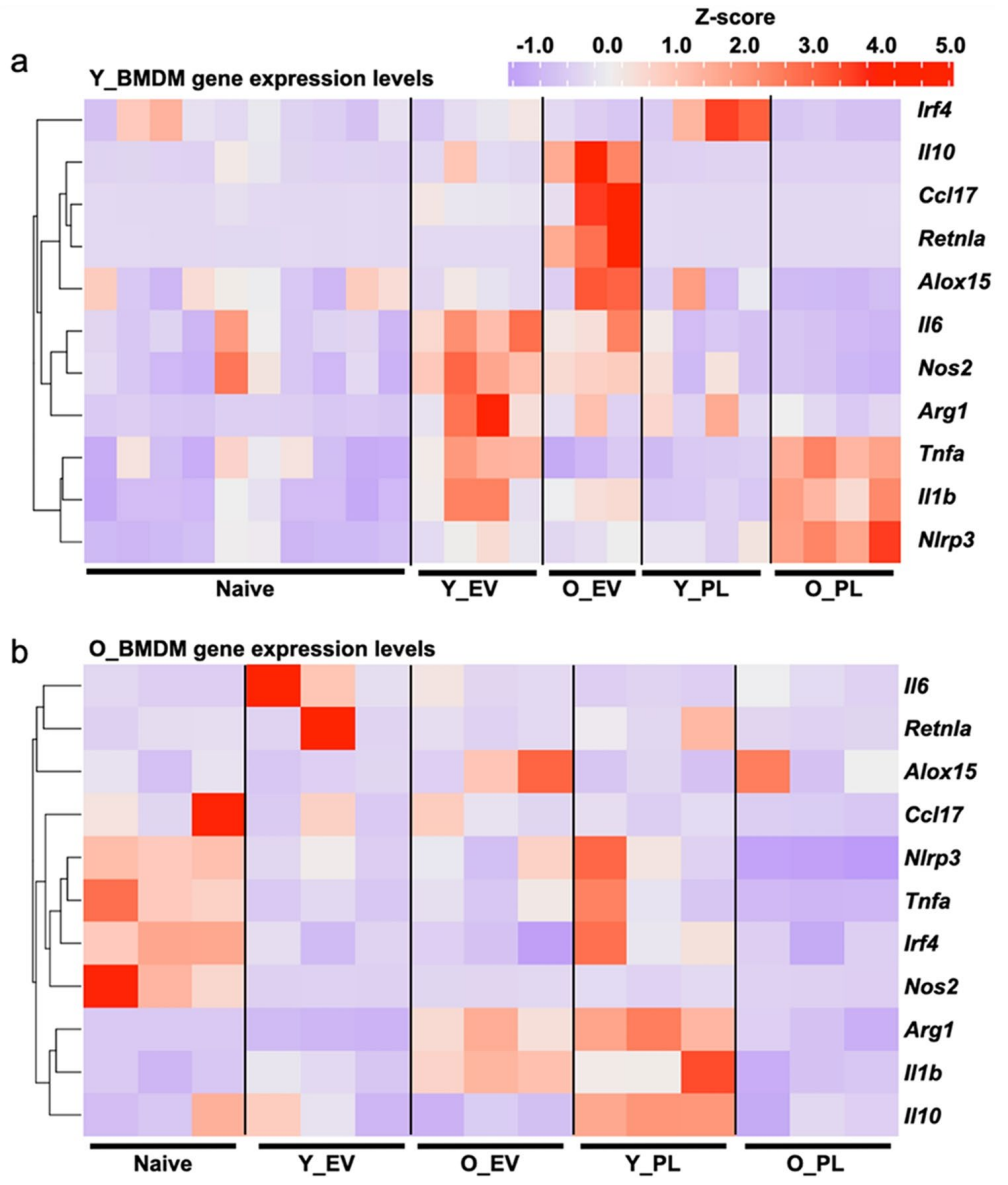
Hierarchical clustering of BMDM gene expression across all experimental conditions (**a**) Heatmap showing Z-scores of normalised (*mRNA/Hnrnpa1*) gene expression in naïve and treated Y_BMDMs. Hierarchical clustering is indicated on the left. **b**) Heatmap for O_BMDMs. Both heatmaps are presented using the same colour scale, shown in the upper right. Y_EV = young mouse plasma EV, O_EV = old mouse plasma EV, Y_PL = young mouse plasma, O_PL = old mouse plasma

To summarise the BMDM gene expression data from all the experimental conditions and to allow for simple visual and statistical comparisons, we used hierarchical clustering to generate a heatmap of gene expression values, normalised to *Hnrnpa1* and expressed as Z-scores. The heatmap for Y_BMDMs (Fig. 7a) clearly shows clusters such as higher expression of M1 (*Nlrp3*,* Tnfa*,* Il1b*) markers with O_PL and higher expression of M2 markers (*Il10*, *Retnla*, *Ccl17*) by O_EVs. In Fig. 7b, higher baseline M1 marker expression by O_BMDMs (*Tnfa*,* Nos2*) is also notable, as is the neutral effect of O_PL.

## Conclusion

In conclusion, this study revealed age-related differences in the effects of EVs on macrophage function, with notable variations in inflammatory and anti-inflammatory markers. While EVs from young mice tended towards suppressing inflammatory markers, neither young nor old EVs induced a clear macrophage polarisation phenotype. This was contrary to plasma from old mice which was distinctly pro-inflammatory. This shows that EVs are a separate compartment of circulating factors. In addition, analysis of EV miRNA cargo found very few significant differences between young and old donors, and we did not replicate results published by others. More research is needed to examine the specific interactions between EVs and EV cargo with other cellular components of the ageing phenotype.

## Discussion

The results of this work reported that older mice have a lower concentration of plasma EVs, with smaller average diameters, which agrees with previous studies [23, 25]. We also found baseline differences in naïve Y/O_BMDMs, with O_BMDMs having a tendency towards higher pro-inflammatory expression. When combining Y_ and O_ mouse plasma with Y_/O_BMDMs, we found that old mouse plasma was markedly pro-inflammatory when incubated with Y_BMDMs. This may be attributable to higher levels of pro-inflammatory cytokines, which are well-described in ageing [16]. Both young and old plasma (Y/O_PL) reduced *Nos2* expression in O_BMDMs, and Y_PL additionally increased *Arg1*. In age-matched treatments (i.e. Y_PL with Y_BMDMs) the effects were less pronounced. These results show that donor age affects both plasma and BMDMs, with older donors having a more inflammatory phenotype. However, when we examined EVs isolated from plasma samples, the results were much less definitive; neither Y_ EVs nor O_EVs stimulated clear polarisation in naïve BMDMs. EVs from both young and old mice altered the response of BMDMs to LPS-induced stimulation, lowering *Il6* and raising *Il10*, indicating a degree of anti-inflammatory function; however, there was no difference between Y_EV and O_EVs. Together, our results indicate that plasma EVs appear to play age-dependent roles in immunomodulation, but this is far more complex than “old = pro-inflammatory” or “young = anti-inflammatory”.

When interpreting the results of our study, there are some methodological factors which should be considered. Firstly, we chose to use young (7–12 week old) and old (70–90 week old) adult mice rather than juvenile/adolescent (< 5 week old), or geriatric (> 100 week old) animals. This decision may have resulted in less differences in EV function or cargo being detected, but we believed this was the most relevant comparison which is also in line with other published studies of young/aged mice [23]. All mice were of the C57BL/6 strain, since they are well-characterised and widely-used in ageing research. Additionally, the mice in our study were purchased from the same national animal supplier and were housed in the same facility under the same conditions to reduce variability. Mice were obtained in several separate batches and experiments were repeated multiple times to obtain the results, thus minimising any batch-dependent effects. We chose to use primary BMDMs to study polarisation rather than cell lines such as RAW264.7, since the latter displays more limited responses to polarising stimuli than primary macrophages. We also used mouse EVs and mouse BMDMs to avoid potential xenobiotic incompatibilities between EV cargo and recipient cells. However, this comes with the caveat that some of our results may not reflect human biology. Secondly, it is increasingly understood that EV isolation methods can capture different sub-populations of EVs, and co-isolate different proteins, which in turn affects their measured cargo [52]. There is no single gold-standard method for EV isolation, with each method having its own advantages and disadvantages [53, 54]. For this study, we elected to use plasma precipitation-based methods for EV isolation, allowing us to achieve higher yields from relatively small samples. This method is widely used in the field and has been extensively validated, but it does result in significantly more non-EV protein co-isolation than other techniques such as size exclusion chromatography [55, 56]. Nevertheless, our cryoEM, NTA and the particle: protein ratio data all indicated successful EV isolation, and we observed clear and large differences in function of the purified EV population compared to unpurified whole plasma. The role of EV co-isolated proteins and nucleic acids is unclear, and recent evidence suggests that they may be constituents of a biologically active EV corona, particularly in circulation [57]. Using precipitation, we were able to isolate plasma EVs, and detected multiple expected plasma EV miRNAs based on online datasets (Vesiclepedia, EV-track etc.) Similarly, all samples were handled using the same techniques, thus any differences between samples in our study should be attributed to mouse age rather than isolation methods. However, the differences to other studies may be due to EV isolation methods.

In terms of plasma EV miRNA cargo, we found only minor differences between young and old mice, which is contrary to some previous studies which have identified many differences [23, 27]. Our spike-in miRNA data and a comparison of housekeeping miRNA expression demonstrated that our miRNA arrays were extremely consistent, with less than 0.1-fold changes between control wells in any two plates. Our research group also has previous experience using this kit for EV miRNA profiling [29, 39]. Thus, we are confident that any detected differences (or lack thereof) should be attributable to biological factors rather than assay or operator variability. Although our sample size was relatively small, other studies using the same sample size have identified differences in circulating EV miRNAs [27]. Three mice per group would be sufficient to detect any gross global changes which are truly conserved with ageing, particularly when the strain of mice is matched. For example, Lee and colleagues found significantly higher miR-500 and miR-770 in aged rat serum EVs compared to young rats (*n* = 3 per group); EVs were also isolated using precipitation methods and were an average size of 100 nm in their study [27]. However, in our data, both miR-500 (mean CT 36.0, *P* = 0.40) and miR-770 (CT 34.4, *P* = 0.59) were found at very low levels and were not different between young and old animals. Similarly, they found a significant reduction of miR-450a-5p, but we found very low levels of this miRNA (mean CT 35.8) and no significant changes with age (*P* = 0.58). In another study analysing serum EV miRNA cargo from 3-, 8- and 12-month-old male C57BL/6 mice (*n* = 3 per group), the researchers reported significant upregulation of miR-184-3p and miR-200b-5p, and downregulation of miR-126b-5p and 466c-5p with increased ageing [58]. In comparison, our samples showed no changes in miR-184-3p expression (mean CT 35.3), and a non-significant increase in miR-200b-5p (mean CT 34.2 and 33.2 in young and old mice respectively), while mmu-miR-466c-5p was detected in only one young mice sample, at a low concentration (CT 36.45). Our analysis panel did not contain the miR-126b sequence. Another team of researchers using miRNA array chips showed different serum miRNA expression profiles in young and old C57BL/6 mice, with none of the aforementioned sequences appearing to be significantly different [59]. The data in our current study revealed some similar and some opposite trends in the up- and downregulation of the sequences (e.g., similarly upregulated miR-215-5p, miR-194-5p, and downregulated let-7a-5p, let-7f-5p, 103-3p and others), however none of them were statistically significant. The large differences in expression may be because we analysed plasma rather than serum, since serum includes EV released from platelets which carry their own cargo [9, 60]. However, from the biological point-of-view, this implies that these circulating factors are not be universally altered in circulation with ageing, thus their true biological significance is unknown. Future work could explore the roles of the miRNAs and their effects on macrophages.

As mentioned in the results section, Alibhai and colleagues profiled plasma EV miRNAs from similarly aged C57BL6 mice (3 months vs. 18–21-month, *n* = 4 per group) as our study, also using a qPCR-based panel [23]. Both of our studies reported reduced EV particle counts and smaller EV size in aged animals, suggesting this is a true biological phenomenon. We also both showed anti-inflammatory effects of EVs from young mice and we both measured consistent, stable, high expression of miR-16, miR-126 and miR-150 in plasma EVs from both young and old animals. This indicates no major differences in the quality of EV isolation or sample handling between our two studies. However, our current study did not find the changes in miR-146a, miR-21 or let-7a which were reported in their study. One difference is that our study used male mice, whereas their study used female mice; and sex differences in circulating miRNAs have been previously described [61]. The largest change we detected (increased miR-192-3p) was also not reported in the Alibhai study, but another study of young and old male C57BL/6JrSlc mice singled out miR-192 as an essential ageing marker with anti-inflammatory effect on macrophages [62]. Wang and colleagues’ study showed mmu-miR-192-**5p** as significantly upregulated in old mice, while it was similarly expressed in our young and old mice samples. As both 5p and 3p sequences originate from the same precursor, it is unknown what affects the different expressions of the mature miRNAs and how coordinated are their regulatory actions in target cells. A very recent study by Chen and colleagues also examined mouse plasma miRNAs in ageing, and found increased miR-29a-3p and miR-34a-5p and decreased miR-144-3p, miR-149-5p and miR-455-3p [26]. In our data, none of the same increases were detected (shown in **Supplemental Table 2**) but we did find similar decreases, though they did not meet statistical significance once corrected for multiple comparisons. Together, the results of our study and others’ illustrate the heterogeneity of EV miRNA cargo. This may also reflect methodological variables in animals, EV isolation methods, miRNA quantification, statistical analyses, or other parameters. Thus, we suggest each study should each be interpreted within the context of their own methodological parameters and experimental designs. However, as a broader point, we believe that if these EV miRNAs are not consistently found across slightly different methodologies in highly-controlled laboratory settings using inbred mice, it inherently raises questions about their true biological relevance in ageing. There is a large body of evidence showing therapeutic value of EVs, and their importance in health homeostasis and disease progression. However, links between EV subtype, cargo and recipient cell responses remain unclear. Further studies are needed to determine whether identified changes with ageing are consistent across different species, sexes and experimental models. Only then can their true biological significance be understood.

## Electronic supplementary material

Below is the link to the electronic supplementary material.

Supplementary Material 1

## Data Availability

The datasets supporting the conclusions of this article are included within the article and its additional files.

## References

[1] Welsh JA, Goberdhan DCI, O’Driscoll L, Buzas EI, Blenkiron C, Bussolati B, et al. Minimal information for studies of extracellular vesicles (MISEV2023): from basic to advanced approaches. J Extracell Vesicles. 2024. 10.1002/jev2.12404. 38326288 10.1002/jev2.12404 PMC10850029

[2] Ilvonen P, Pusa R, Härkönen K, Laitinen S, Impola U. Distinct targeting and uptake of platelet and red blood cell-derived extracellular vesicles into immune cells. J Extracell Biology. 2024. 10.1002/jex2.130. 10.1002/jex2.130 PMC11080822 38938679

[3] Driedonks T, Jiang L, Carlson B, Han Z, Liu G, Queen SE, et al. Pharmacokinetics and biodistribution of extracellular vesicles administered intravenously and intranasally to Macaca nemestrina. J Extracell Biology. 2022;1:1–34. 10.1002/jex2.59 PMC9799283 36591537

[4] Waury K, Gogishvili D, Nieuwland R, Chatterjee M, Teunissen CE, Abeln S. (2024). Proteome encoded determinants of protein sorting into extracellular vesicles. J Extracell Biology, 3:2023.02.01.526570. 10.1002/jex2.120 PMC11080751 38938677

[5] Biemmi V, Milano G, Ciullo A, Cervio E, Burrello J, Cas MD, et al. Inflammatory extracellular vesicles prompt heart dysfunction via TRL4-dependent NF-κB activation. Theranostics. 2020;10:2773–90. 32194834 10.7150/thno.39072 PMC7052909

[6] Luo Z, Hu X, Wu C, Chan J, Liu Z, Guo C, et al. Plasma exosomes generated by ischaemic preconditioning are cardioprotective in a rat heart failure model. Br J Anaesth. 2023;130:29–38. 36347723 10.1016/j.bja.2022.08.040 PMC9875906

[7] Abdelmohsen K, Herman AB, Carr AE, Henry-Smith CA, Rossi M, Meng Q, et al. Survey of organ‐derived small extracellular vesicles and particles (sEVPs) to identify selective protein markers in mouse serum. J Extracell Biology. 2023. 10.1002/jex2.106. 10.1002/jex2.106 PMC10512735 37744304

[8] Nieuwland R, Siljander PRM. A beginner’s guide to study extracellular vesicles in human blood plasma and serum. J Extracell Vesicles doi. 2024. 10.1002/jev2.12400. 10.1002/jev2.12400 PMC10775135 38193375

[9] Małys MS, Köller MC, Papp K, Aigner C, Dioso D, Mucher P, et al. Small extracellular vesicles are released ex vivo from platelets into serum and from residual blood cells into stored plasma. J Extracell Biology. 2023. 10.1002/jex2.88. 10.1002/jex2.88 PMC11080719 38938276

[10] Max KEA, Bertram K, Akat KM, Bogardus KA, Li J, Morozov P, et al. Human plasma and serum extracellular small RNA reference profiles and their clinical utility. Proc Natl Acad Sci U S A. 2018;115:E5334–43. 29777089 10.1073/pnas.1714397115 PMC6003356

[11] Wang H, Maimaitiaili R, Yao J, Xie Y, Qiang S, Hu F, et al. Percutaneous intracoronary delivery of plasma extracellular vesicles protects the myocardium against Ischemia-Reperfusion Injury in Canis. Hypertension; 2021. pp. 1541–54. 10.1161/HYPERTENSIONAHA.121.17574 34488435

[12] Vicencio JM, Yellon DM, Sivaraman V, Das D, Boi-Doku C, Arjun S, et al. Plasma exosomes protect the myocardium from ischemia-reperfusion injury. J Am Coll Cardiol. 2015;65:1525–36. 25881934 10.1016/j.jacc.2015.02.026

[13] Davidson SM, Andreadou I, Barile L, Birnbaum Y, Cabrera-Fuentes HA, Cohen MV, et al. Circulating blood cells and extracellular vesicles in acute cardioprotection. Cardiovasc Res. 2019;115:1156–66. 30590395 10.1093/cvr/cvy314 PMC6529916

[14] Adamczyk AM, Leicaj ML, Fabiano MP, Cabrerizo G, Bannoud N, Croci DO, et al. Extracellular vesicles from human plasma dampen inflammation and promote tissue repair functions in macrophages. J Extracell Vesicles. 2023. 10.1002/jev2.12331. 37272889 10.1002/jev2.12331 PMC10241174

[15] Plowden J, Renshaw-Hoelscher M, Engleman C, Katz J, Sambhara S. Innate immunity in aging: impact on macrophage function. Aging Cell. 2004;3:161–7. 15268749 10.1111/j.1474-9728.2004.00102.x

[16] López-Otín C, Blasco MA, Partridge L, Serrano M, Kroemer G. The hallmarks of aging. Cell. 2013;153:1194–217. 23746838 10.1016/j.cell.2013.05.039 PMC3836174

[17] Pandika M. Looking to Young blood to treat the diseases of Aging. ACS Cent Sci. 2019;5:1481–4. 31572771 10.1021/acscentsci.9b00902 PMC6764071

[18] Villeda SA, Plambeck KE, Middeldorp J, Castellano JM, Mosher KI, Luo J, et al. Young blood reverses age-related impairments in cognitive function and synaptic plasticity in mice. Nat Med. 2014;20:659–63. 24793238 10.1038/nm.3569 PMC4224436

[19] Gan KJ, Südhof TC. Specific factors in blood from young but not old mice directly promote synapse formation and NMDA-receptor recruitment. Proc Natl Acad Sci U S A. 2019;116:12524–33. 31160442 10.1073/pnas.1902672116 PMC6589664

[20] Horowitz AM, Fan X, Bieri G, Smith LK, Sanchez-Diaz CI, Schroer AB, et al. Blood factors transfer beneficial effects of exercise on neurogenesis and cognition to the aged brain. Sci (1979). 2020;369:167–73. 10.1126/science.aaw2622 PMC7879650 32646997

[21] Wei S-Y, Chou Y-H, Chang F-C, Huang S-Y, Lai C-F, Lin S-L. Young plasma attenuated chronic kidney Disease Progression after Acute kidney Injury by inhibiting inflammation in mice. Aging Dis. 2024. 10.14336/AD.2023.1230. 38421825 10.14336/AD.2023.1230 PMC11567270

[22] Liu D, Lun L, Huang Q, Ning Y, Zhang Y, Wang L, et al. Youthful systemic milieu alleviates renal ischemia-reperfusion injury in elderly mice. Kidney Int. 2018;94:268–79. 29935950 10.1016/j.kint.2018.03.019

[23] Alibhai FJ, Lim F, Yeganeh A, DiStefano PV, Binesh-Marvasti T, Belfiore A, et al. Cellular senescence contributes to age-dependent changes in circulating extracellular vesicle cargo and function. Aging Cell. 2020. 10.1111/acel.13103. 31960578 10.1111/acel.13103 PMC7059145

[24] Yin Y, Chen H, Wang Y, Zhang L, Wang X. Roles of extracellular vesicles in the aging microenvironment and age-related diseases. J Extracell Vesicles. 2021. 10.1002/jev2.12154. 34609061 10.1002/jev2.12154 PMC8491204

[25] Zhang H, Lin S, Mcelroy CL, Wang B, Jin D, Uteshev VV, et al. Circulating pro-inflammatory exosomes worsen stroke outcomes in aging. Circ Res. 2021;129:121–40. 10.1161/CIRCRESAHA.121.318897 PMC8448978 34399581

[26] Chen X, Luo Y, Zhu Q, Zhang J, Huang H, Kan Y, et al. Small extracellular vesicles from young plasma reverse age-related functional declines by improving mitochondrial energy metabolism. Nat Aging Doi. 2024. 10.1038/s43587-024-00612-4. 10.1038/s43587-024-00612-4 PMC11186790 38627524

[27] Lee EK, Jeong HO, Bang EJ, Kim CH, Mun JY, Noh S, et al. The involvement of serum exosomal mir-500-3p and miR-770- 3p in aging: modulation by calorie restriction. Oncotarget. 2018;9:5578–87. 29464019 10.18632/oncotarget.23651 PMC5814159

[28] Liu JR, Cai GY, Ning YC, Wang JC, Lv Y, Guo YN, et al. Caloric restriction alleviates aging-related fibrosis of kidney through downregulation of miR-21 in extracellular vesicles. Aging. 2020;12:18052–72. 32963130 10.18632/aging.103591 PMC7585074

[29] Jaimes MSV, Liao C, Chen MM, Czosseck A, Lee T, Chou Y, et al. Assessment of circulating extracellular vesicles from calorie-restricted mice and humans in ischaemic injury models. J Extracell Biology. 2023. 10.1002/jex2.86. 10.1002/jex2.86 PMC11080834 38938283

[30] Chen S, Saeed AFUH, Liu Q, Jiang Q, Xu H, Xiao GG, et al. Macrophages in immunoregulation and therapeutics. Signal Transduct Target Ther doi. 2023. 10.1038/s41392-023-01452-1. 10.1038/s41392-023-01452-1 PMC10200802 37211559

[31] Orecchioni M, Ghosheh Y, Pramod AB, Ley K. Macrophage polarization: different gene signatures in M1(Lps+) vs. classically and M2(LPS-) vs. alternatively activated macrophages. Front Immunol. 2019;10:1–14. 31178859 10.3389/fimmu.2019.01084 PMC6543837

[32] Shi Y, Luo P, Wang W, Horst K, Bläsius F, Relja B, et al. M1 but Not M0 Extracellular vesicles induce polarization of RAW264.7 Macrophages Via the TLR4-NFκB pathway in Vitro. Inflammation. 2020;43:1611–9. 32323096 10.1007/s10753-020-01236-7 PMC7476919

[33] Lv LL, Feng Y, Wu M, Wang B, Li ZL, Zhong X, et al. Exosomal miRNA-19b-3p of tubular epithelial cells promotes M1 macrophage activation in kidney injury. Cell Death Differ. 2020;27:210–26. 31097789 10.1038/s41418-019-0349-y PMC7206053

[34] Gao F, Kataoka M, Liu N, Liang T, Huang ZP, Gu F, et al. Therapeutic role of miR-19a/19b in cardiac regeneration and protection from myocardial infarction. Nat Commun Doi. 2019. 10.1038/s41467-019-09530-1. 10.1038/s41467-019-09530-1 PMC6470165 30996254

[35] Yang HC, Rossini M, Ma LJ, Zuo Y, Ma J, Fogo AB. Cells derived from young bone marrow alleviate renal aging. J Am Soc Nephrol. 2011;22:2028–36. 21965376 10.1681/ASN.2010090982 PMC3231782

[36] Toda G, Yamauchi T, Kadowaki T, Ueki K. Preparation and culture of bone marrow-derived macrophages from mice for functional analysis. STAR Protoc. 2021;2:100246. 33458708 10.1016/j.xpro.2020.100246 PMC7797923

[37] Mendoza R, Banerjee I, Manna D, Reghupaty SC, Yetirajam R, Sarkar D. Mouse bone marrow cell isolation and macrophage differentiation. Methods Mol Biol. 2022;2455:85–91. 35212988 10.1007/978-1-0716-2128-8_8 PMC8936184

[38] Czosseck A, Chen MM, Nguyen H, Meeson A, Hsu C, Chen C, et al. Porous scaffold for mesenchymal cell encapsulation and exosome-based therapy of ischemic diseases. J Controlled Release. 2022;352:879–92. 10.1016/j.jconrel.2022.10.057 36370875

[39] Livkisa D, Chang T, Burnouf T, Czosseck A, Le NTN, Shamrin G, et al. Extracellular vesicles purified from serum-converted human platelet lysates offer strong protection after cardiac ischaemia/reperfusion injury. Biomaterials. 2024;306:122502. 38354518 10.1016/j.biomaterials.2024.122502

[40] Eitan E, Green J, Bodogai M, Mode NA, Bæk R, Jørgensen MM, et al. Age-related changes in plasma extracellular vesicle characteristics and internalization by Leukocytes. Sci Rep. 2017;7:1–14. 28465537 10.1038/s41598-017-01386-z PMC5430958

[41] Brennan K, Martin K, FitzGerald SP, O’Sullivan J, Wu Y, Blanco A, et al. A comparison of methods for the isolation and separation of extracellular vesicles from protein and lipid particles in human serum. Sci Rep. 2020;10:1–13. 31974468 10.1038/s41598-020-57497-7 PMC6978318

[42] Li WJ, Chen H, Tong ML, Niu JJ, Zhu XZ, Lin LR. Comparison of the yield and purity of plasma exosomes extracted by ultracentrifugation, precipitation, and membrane-based approaches. Open Chem. 2022;20:182–91.

[43] Webber J, Clayton A. How pure are your vesicles? J Extracell Vesicles. 2013;2:1–6. 10.3402/jev.v2i0.19861 PMC3760653 24009896

[44] Liu S, Chen J, Shi J, Zhou W, Wang L, Fang W, et al. M1-like macrophage-derived exosomes suppress angiogenesis and exacerbate cardiac dysfunction in a myocardial infarction microenvironment. Basic Res Cardiol. 2020. 10.1007/s00395-020-0781-7. 32112145 10.1007/s00395-020-0781-7

[45] Shih WC, Jang IH, Kruglov V, Dickey D, Cholensky S, Bernlohr DA, et al. Role for BLT1 in regulating inflammation within adipose tissue immune cells of aged mice. Immun Ageing. 2024. 10.1186/s12979-024-00461-0. 39187841 10.1186/s12979-024-00461-0 PMC11346001

[46] Tanaka A, To J, O’Brien B, Donnelly S, Lund M. Selection of reliable reference genes for the normalisation of gene expression levels following time course LPS stimulation of murine bone marrow derived macrophages. BMC Immunol. 2017. 10.1186/s12865-017-0223-y. 28974200 10.1186/s12865-017-0223-y PMC5627409

[47] Gámez-Valero A, Campdelacreu J, Vilas D, Ispierto L, Reñé R, Álvarez R, et al. Exploratory study on microRNA profiles from plasma-derived extracellular vesicles in Alzheimer’s disease and dementia with Lewy bodies. Transl Neurodegener. 2019. 10.1186/s40035-019-0169-5. 31592314 10.1186/s40035-019-0169-5 PMC6775659

[48] Pu C, Huang H, Wang Z, Zou W, Lv Y, Zhou Z, et al. Extracellular vesicle-associated mir-21 and mir-144 are markedly elevated in serum of patients with hepatocellular carcinoma. Front Physiol. 2018. 10.3389/fphys.2018.00930. 30065664 10.3389/fphys.2018.00930 PMC6056643

[49] Lassen TR, Just J, Hjortbak MV, Jespersen NR, Stenz KT, Gu T, et al. Cardioprotection by remote ischemic conditioning is transferable by plasma and mediated by extracellular vesicles. Basic Res Cardiol. 2021;116:16. 33689033 10.1007/s00395-021-00856-w

[50] Yamada K, Takizawa S, Ohgaku Y, Asami T, Furuya K, Yamamoto K, et al. MicroRNA 16-5p is upregulated in calorie-restricted mice and modulates inflammatory cytokines of macrophages. Gene. 2020;725:144191. 31654705 10.1016/j.gene.2019.144191

[51] Rosenberger CM, Podyminogin RL, Diercks AH, Treuting PM, Peschon JJ, Rodriguez D, et al. miR-144 attenuates the host response to influenza virus by targeting the TRAF6-IRF7 signaling axis. PLoS Pathog. 2017. 10.1371/journal.ppat.1006305. 28380049 10.1371/journal.ppat.1006305 PMC5393898

[52] Teng F, Fussenegger M. Shedding light on Extracellular Vesicle Biogenesis and Bioengineering. Adv Sci. 2021;8:1–17. 10.1002/advs.202003505 PMC7788585 33437589

[53] Benayas B, Morales J, Egea C, Armisén P, Yáñez-Mó M. Optimization of extracellular vesicle isolation and their separation from lipoproteins by size exclusion chromatography. J Extracell Biology. 2023. 10.1002/jex2.100. 10.1002/jex2.100 PMC11080862 38939075

[54] Lai JJ, Chau ZL, Chen SY, Hill JJ, Korpany KV, Liang NW, et al. Exosome Processing and characterization approaches for Research and Technology Development. Advanced Science; 2022. pp. 1–93. 10.1002/advs.202103222 PMC9130923 35332686

[55] Fernández-Rhodes M, Adlou B, Williams S, Lees R, Peacock B, Aubert D, et al. Defining the influence of size‐exclusion chromatography fraction window and ultrafiltration column choice on extracellular vesicle recovery in a skeletal muscle model. J Extracell Biology. 2023. 10.1002/jex2.85. 10.1002/jex2.85 PMC11080914 38939692

[56] Helwa I, Cai J, Drewry MD, Zimmerman A, Dinkins MB, Khaled ML, et al. A comparative study of serum exosome isolation using differential ultracentrifugation and three commercial reagents. PLoS ONE. 2017;12:1–22. 10.1371/journal.pone.0170628 PMC5256994 28114422

[57] Tóth E, Turiák L, Visnovitz T, Cserép C, Mázló A, Sódar BW, et al. Formation of a protein corona on the surface of extracellular vesicles in blood plasma. J Extracell Vesicles. 2021. 10.1002/jev2.12140. 34520123 10.1002/jev2.12140 PMC8439280

[58] Lee BR, Kim JH, Choi ES, Cho JH, Kim E. Effect of young exosomes injected in aged mice. Int J Nanomed. 2018;13:5335–45. 10.2147/IJN.S170680 PMC6141108 30254438

[59] Wang W, Wang L, Ruan L, Oh J, Dong X, Zhuge Q, et al. Extracellular vesicles extracted from young donor serum attenuate inflammaging via partially rejuvenating aged T-cell immunotolerance. FASEB J. 2018;32:5899–912. 10.1096/fj.201800059R PMC6181631 29782203

[60] Burnouf T, Chou M-L, Lundy DJ, Chuang E-Y, Tseng C-L, Goubran H. Expanding applications of allogeneic platelets, platelet lysates, and platelet extracellular vesicles in cell therapy, regenerative medicine, and targeted drug delivery. J Biomed Sci. 2023;30:79. 37704991 10.1186/s12929-023-00972-w PMC10500824

[61] Noren Hooten N, Byappanahalli AM, Vannoy M, Omoniyi V, Evans MK. Influences of age, race, and sex on extracellular vesicle characteristics. Theranostics. 2022;12:4459–76. 35673574 10.7150/thno.72676 PMC9169362

[62] Tsukamoto H, Kouwaki T, Oshiumi H. Aging-Associated Extracellular vesicles contain Immune Regulatory microRNAs alleviating Hyperinflammatory State and Immune Dysfunction in the Elderly. iScience. 2020. 10.1016/j.isci.2020.101520. 32927264 10.1016/j.isci.2020.101520 PMC7495115

