# Supplementary material for: Distinct immunomodulation elicited by young versus aged extracellular vesicles in bone marrow-derived macrophages
Source: Immun Ageing. 2024 Oct 21;21:72. doi: 10.1186/s12979-024-00472-x (PMC11492788; doi:10.1186/s12979-024-00472-x)
Supplement: Supplementary file 1 — Supplementary Material 1 [file 12979_2024_472_MOESM1_ESM.docx]

**Supporting information**

**Distinct Immunomodulation Elicited by Young Versus Aged Extracellular Vesicles in Bone Marrow-Derived Macrophages**

Dora Livkisa^1^, Tsung-Lin Lee^2^, Wei-Ting Yeh^3^, Manuel S.V. Jaimes^4^, Barbara Szomolay^5,6^, Chia-Te Liao^2,7,8^* & David J. Lundy^1,4,9^*

1. International PhD Program in Biomedical Engineering, College of Biomedical Engineering, Taipei Medical University, 301 Yuantong Road, New Taipei City 235603, Taiwan
2. Division of Nephrology, Department of Internal Medicine, Shuang Ho Hospital, Taipei Medical University, New Taipei City 235603, Taiwan
3. School of Biomedical Engineering, Taipei Medical University, 301 Yuantong Road, New Taipei City 235603, Taiwan
4. Graduate Institute of Biomedical Materials & Tissue Engineering, College of Biomedical Engineering, Taipei Medical University, 301 Yuantong Road, New Taipei City 235603, Taiwan
5. Systems Immunity Research Institute, Cardiff University School of Medicine, Cardiff, United Kingdom
6. Division of Infection and Immunity, Cardiff University School of Medicine, Cardiff, United Kingdom
7. Division of Nephrology, Department of Internal Medicine, School of Medicine, College of Medicine, Taipei Medical University, 250 Wuxing Street, Taipei 110, Taiwan
8. Taipei Medical University-Research Center of Urology and Kidney, Taipei Medical University, Taipei 110, Taiwan
9. Cell Therapy Center, Taipei Medical University Hospital, 250 Wuxing Street, Taipei 110, Taiwan

**Correspondence to be addressed to**: Chia-Te Liao or David J. Lundy.

Taipei Medical University, 301 Yuantong Road, New Taipei City, Taiwan.

Email: [ctliao19386@tmu.edu.tw](mailto:ctliao19386@tmu.edu.tw) or [dlundy@tmu.edu.tw](mailto:dlundy@tmu.edu.tw)

**Running title**: Age-dependent exosome immunomodulation

|  | Forward | Reverse |
| --- | --- | --- |
| Housekeeping | | |
| *Hnrnpa1* | CCA ACA CTG GAC GAT CAA GAG G | ATG ACA CGA CCA TCC AGC CTG T |
| *Gapdh* | CAT CAC TGC CAC CCA GAA GAC TG | ATG CCA GTG AGC TTC CCG TTC AG |
| *Stx5a* | CTG AAA CAG CAG AGG AAC CGT C | GGT CCA TCA TGT CAA TAG CCA CG |
| Polarization markers | | |
| *Il1b* | TTC AGG CAG GCA GTA TCA CTC | GAA GGT CCA CGG GAA AGA CAC |
| *Nlrp3* | ATT ACC CGC CCG AGA AAG G | CAT GAG TGT GGC TAG ATC CAA G |
| *Il6* | TCT ATA CCA CTT CAC AAG TCG GA | GAA TTG CCA TTG CAC AAC TCT TT |
| *Tnfa* | CAG GCG GTG CCT ATG TCT C | CGA TCA CCC CGA AGT TCA GTA G |
| *Nos2* | GTT CTC AGC CCA ACA ATA CAA GA | GTG GAC GGG TCG ATG TCA C |
| *Arg1* | CTC CAA GCC AAA GTC CTT AGA G | AGG AGC TGT CAT TAG GGA CAT C |
| *Retnla* | CCA ATC CAG CTA ACT ATC CCT CC | ACC CAG TAG CAG TCA TCC CA |
| *Ccl17* | TAC CAT GAG GTC ACT TCA GAT GC | GCA CTC TCG GCC TAC ATT GG |
| *Irf4* | CTT TGA GGA ATT GGT CGA GAG G | GAG AGC CAT AAG GTG CTG TCA |
| *Alox15* | GGC TCC AAC AAC GAG GTC TAC | AGG TAT TCT GAC ACA TCC ACC TT |
| *Il10* | CGG GAA GAC AAT AAC TGC ACC C | CGG TTA GCA GTA TGT TGT CCA GC |
| *Tgfb1* | TGA TAC GCC TGA GTG GCT GTC T | CAC AAG AGC AGT GAG CGC TGA A |
| *Pdl1* | TGC CGA CTA CAA GCG AAT TAC TG | CTG CTT GTC CAG ATG ACT TCG G |

**Supplemental Table 1**

Primers used for assessing bone marrow-derived macrophage (BMDM) polarization. All primers are mouse-specific.

| miRNA ID | Described change | O_EV vs Y_EV log2fc | P |
| --- | --- | --- | --- |
| Our study |  | **Results from our study** | |
| mmu-miR-192-3p |  | 2.763 | 0.017 |
| mmu-miR-877-5p |  | -1.911 | 0.025 |
| mmu-miR-154-5p |  | -2.250 | 0.035 |
| rno-miR-351-5p |  | -1.911 | 0.037 |
| mmu-miR-700-3p |  | 1.352 | 0.048 |
|  |  |  |  |
| PMID 38627524 |  |  |  |
| miR-29a-3p | Increased (~2.5-fold) | -0.081 | 0.948 |
| miR-29c-3p | Increased (~2.5-fold) | -0.310 | 0.823 |
| miR-34a-5p | Increased (~3.0-fold) | - | - |
| miR-144-3p | Decreased (~3.0-fold) | -0.543 | 0.602 |
| miR-149-5p | Decreased (~3.0-fold) | -2.863 | 0.386 |
| miR-455-3p | Decreased (~3.0-fold) | -1.881 | 0.282 |
|  |  |  |  |
| PMID 31960578 | |  | |
| mmu-miR-21a-5p | Increase (~2.5-fold) | -0.131 | 0.896 |
| mmu-miR-22-3p | Increase (~2.5-fold)  Increase (~2.5-fold) | -1.496 | 0.492 |
| mmu-miR-22-5p |  | -1.363 | 0.577 |
| mmu-miR-145a-5p | Increase (~2-fold)  Increase (~2-fold) | -1.104 | 0.503 |
| mmu-miR-145a-3p |  | -1.145 | 0.540 |
| mmu-miR-146a-5p | Increase (~3-fold) | 0.447 | 0.683 |
| mmu-miR-223-3p | Increase (~2.5-fold)  Increase (~2-fold) | 0.359 | 0.726 |
| rno-miR-223-3p |  | 0.348 | 0.731 |
| mmu-let-7a-5p | Increase (~2-fold) | -1.842 | 0.248 |
| mmu-let-7i-5p | Decrease | -0.931 | 0.445 |
| rno-miR-200c-3p | Decrease | -0.185 | 0.887 |
| mmu-miR-455-3p | Decrease  Decrease | -1.881 | 0.283 |
| mmu-miR-455-5p |  | -0.720 | 0.474 |
| mmu-miR-199a-3p | Decrease | -1.823 | 0.370 |
|  |  |  |  |
| PMID 29464019 | |  | |
| miR-770-3p | Increase (165x) | Below threshold | - |
| mmu-miR-500-3p | Increase (223x) | 1.434 | 0.104 |
| miR-6324 | Increase (161x) | Not included | - |
| mmu-miR-455-3p | Increase (132x) | -1.881 | 0.283 |
| rno-miR-450a-5p | Decrease (292x) | -0.835 | 0.584 |
| miR-196c | Decrease (140x) | Below threshold | - |

**Supplemental Table 2**

miRNAs identified in previously-published studies of circulating EV miRNAs in aging. The data shown are from our study. For PMID 31960578, miRNA species (hsa,mmu, rno) was not stated and only the miR name was given (i.e. “miR-145” “miR-21” *etc*.); therefore we show all possible permutations present in our dataset (mmu-miR-145a-3p, mmu-miR-145a-5p, *etc*.) For PMID 29464019, aged rats were investigated and rno-miRNAs were reported.


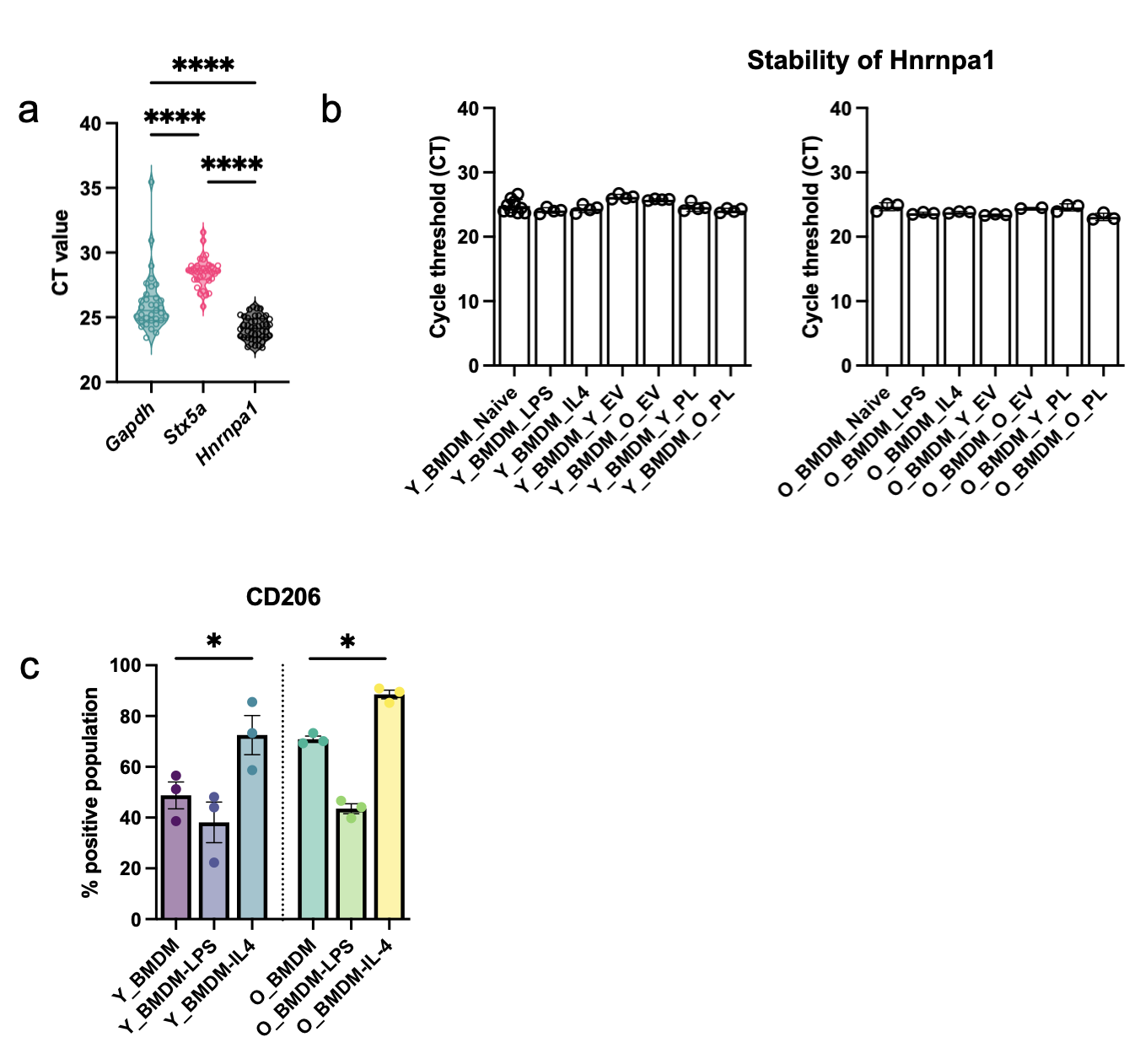

**Supplemental Figure 1**

**Suitability and stability of Hnrnpa1 as a housekeeping gene for bone marrow derived macrophages**

1. Cycle threshold values of *Gapdh*, *Stx5a* and *Hnrnpa1* for all samples. All groups were compared by one-way ANOVA with Tukey’s multiple comparison test.
2. CT values of *Hnrnpa1* for all conditions. Y/O_BMDM = young/old mouse derived bone marrow derived macrophage, Y_EV = young/old mouse derived plasma extracellular vesicle, Y/O_PL = young/old mouse derived plasma.
3. CD206 positive population measured by flow cytometry from Y_BMDM and O_BMDM in naïve, LPS or IL-4-stimulated conditions. Groups were compared to naïve BMDMs by one-way ANOVA with Sidak’s multiple comparison test.


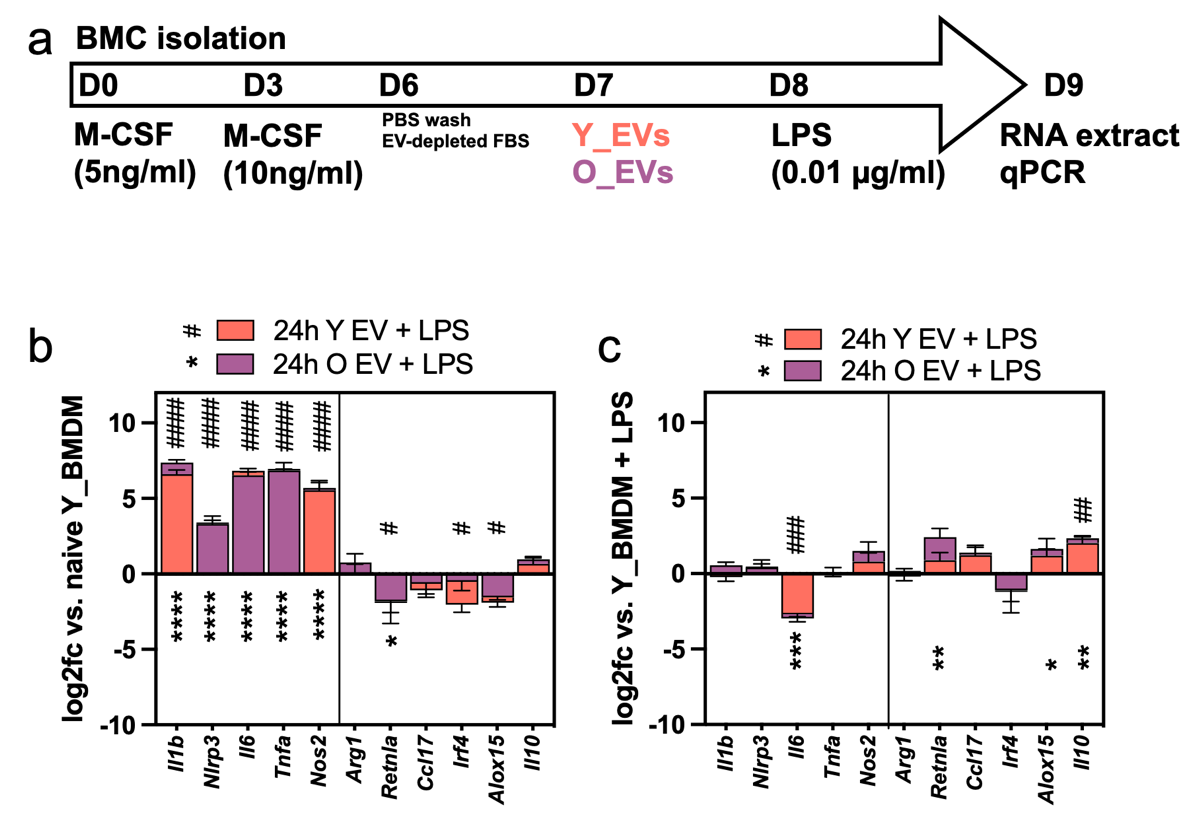


**Supplemental Figure 2**

**Effects of EV pre-treatment on LPS-induced macrophage M1 polarization and Tgfb1, Pdl1 expression**

1. Schematic diagram showing Y_BMC isolation and differentiation to Y_BMDMs, followed by 24h pre-treatment with Y_EVs or O_EVs before 24h low-dose LPS stimulation.
2. Log2 gene expressional changes (log2fc) compared to naïve Y_BMDMs. Comparisons of Y_EVs + LPS vs naive Y_BMDMs are shown by # and O_EVs are shown by *.
3. Changes in gene expression of Y_BMDMs incubated with Y_EVs or O_EVs for 24h, followed by LPS for 24h, compared to Y_BMDMs treated with LPS alone, without EV pre-treatment. Comparisons of Y_EVs vs. LPS Y_BMDMs are shown by # and comparisons of O_EVs vs. LPS Y_BMDMs are shown by *. There were no significant differences between O_EVs and Y_EVs. * = P ≤ 0.05, ** = P < 0.01, *** = P ≤ 0.001, **** = P ≤ 0.0001. All comparisons were performed by two-way ANOVA with Tukey’s post-test applied.


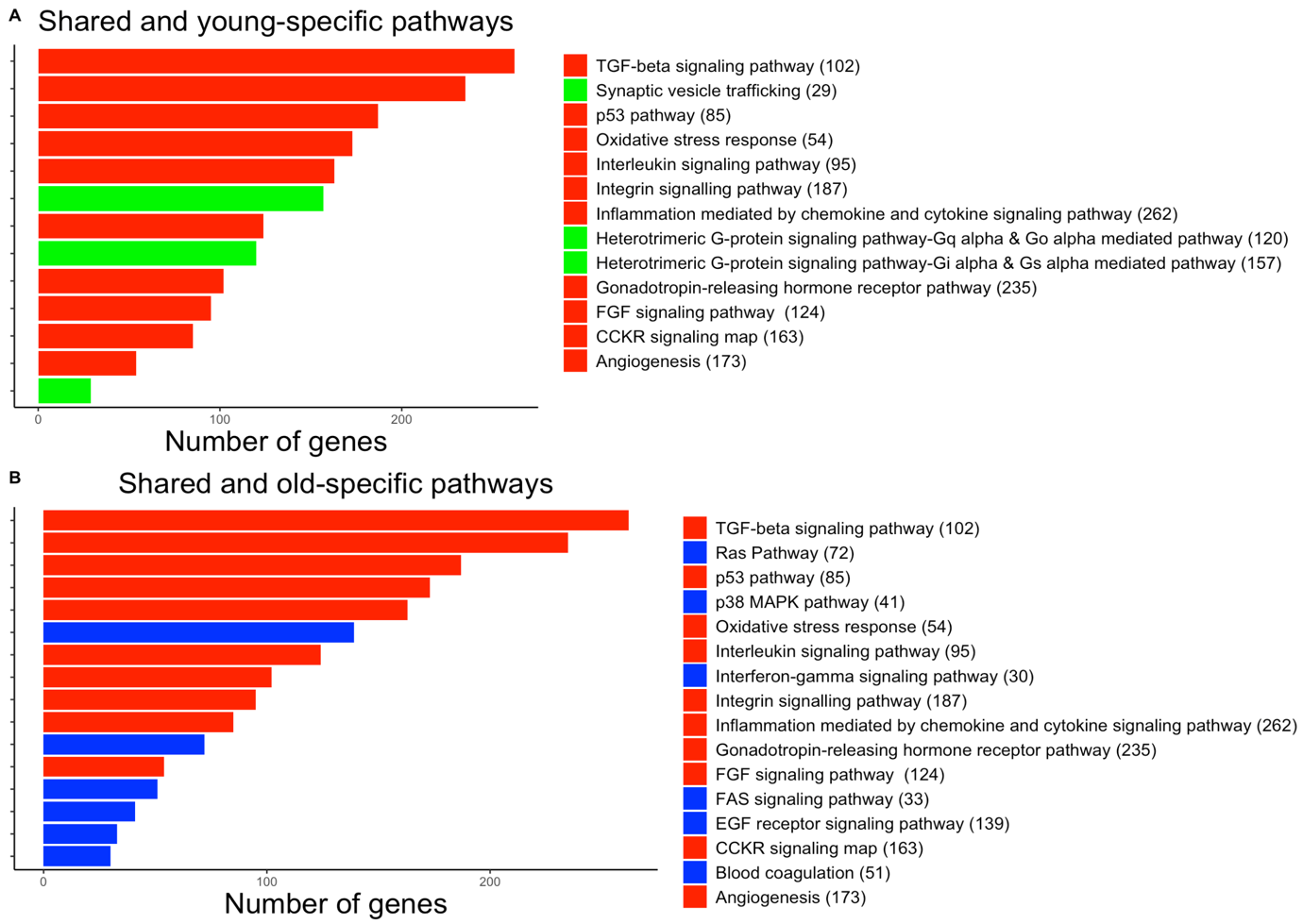


**Supplemental Figure 3**

**mRNA target pathway analysis for top 10 most abundant Y_EV and O_EV miRNAs**.

Out of the top 10 miRNAs, 8 miRNAs had targeting information available for old mice (mmu-miR-144-3p, mmu-miR-19b-3p, mmu-miR-21a-5p, mmu-miR-23a-3p, mmu-miR-24-3p, mmu-miR-25-3p, mmu-miR-3107-5p, rno-miR-223-3p) and for young mice (mmu-miR-126a-3p, mmu-miR-133a-3p, mmu-miR-144-3p, mmu-miR-1a-3p, mmu-miR-21a-5p, mmu-miR-23a-3p, mmu-miR-24-3p, mmu-miR-3107-5p). The microRNA Target Filter results in IPA were filtered based on murine-specific high confidence or experimental observation, targeting 749 mRNAs for old and 718 mRNAs for young, which were used for PANTHER overrepresentation test. Significant pathways (FDR <0.05) are shown. Red indicates shared pathways between young and old EV miRNAs.


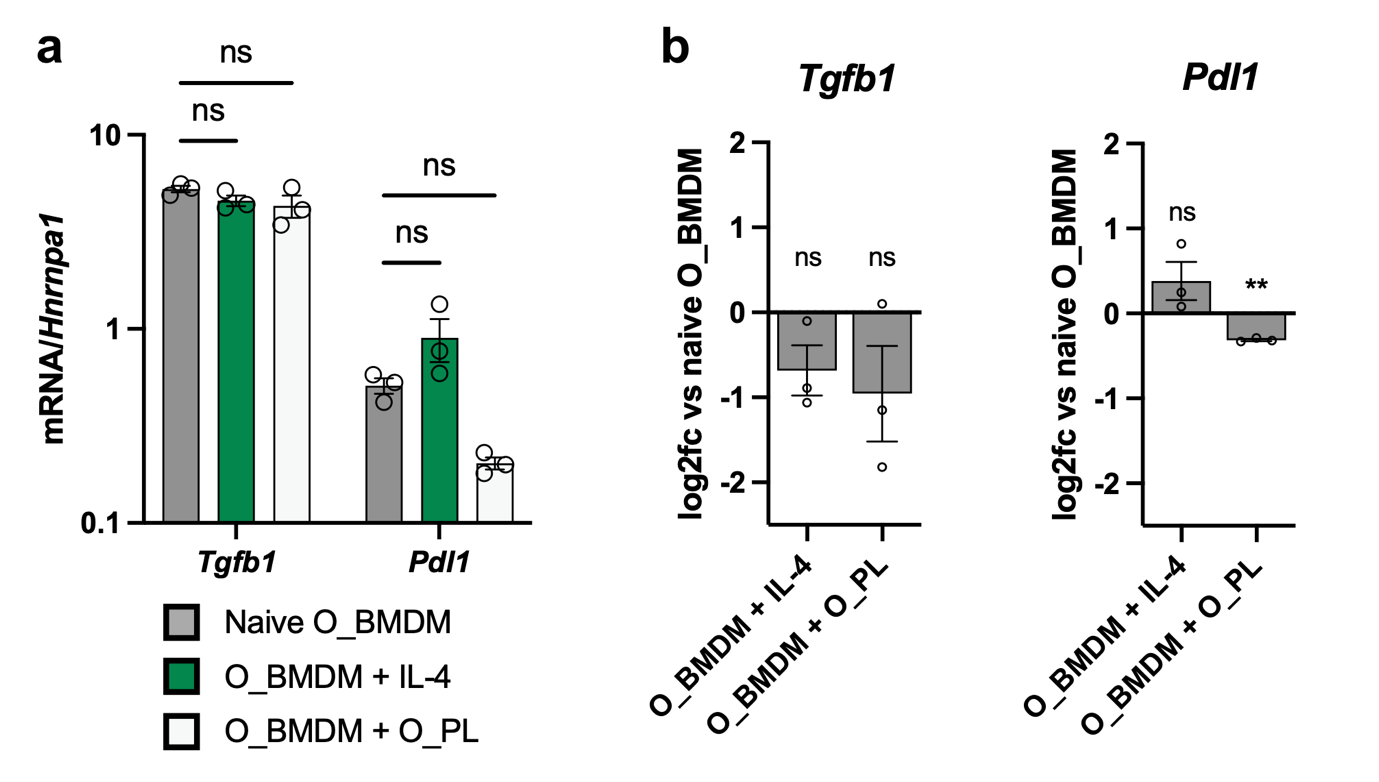


**Supplemental Figure 4**

**Tgfb1 and Pdl1 expression in O_BMDMs**.

1. Expression levels of Tgfb1 and Pdl1 in naïve O_BMDMs and O_BMDMs treated with IL-4 or O_PL. Expression was normalised to *Hnrnpa1*. Samples were compared by two-way ANOVA.
2. *Tgfb1* and *Pdl1* expression shown as log2fc compared to naïve O_BMDMs. Changes were subjected to one-sample t and Wilcoxon tests. ns = not significant (P > 0.05), ** = P ≤ 0.01.
